# FDA approved drug containing acetamide group- a mini review

**DOI:** 10.3389/fchem.2026.1830482

**Published:** 2026-08-07

**Authors:** Sandeep Bindra, Chetana Amrodiya, Sunil Kumar, Della Grace Thomas Parambi, Arun Kumar, Naseer Maliyakkal, Jasha Momo H. Anal, Bijo Mathew

**Affiliations:** 1 Natural Products and Medicinal Chemistry Division, CSIR-Indian Institute of Integrative Medicine, Academy of Scientific and Innovative Research (AcSIR), Ghaziabad, India; 2 Department of Pharmaceutical Chemistry, Amrita School of Pharmacy, Amrita Vishwa Vidyapeetham, Kochi, Kerala, India; 3 Department of Pharmaceutical Chemistry, Faculty of Pharmacy, Jouf University, Sakaka, Saudi Arabia; 4 School of Pharmacy, Sharda University, Knowledge Park III, Greater Noida, India; 5 Department of Anesthesia and Operations, College of Applied Medical Sciences, Khamis Mushait, King Khalid University, Abha, Saudi Arabia

**Keywords:** acetamide-containing drugs, drug-like properties, peptide drugs, toxicity, zanamivir

## Abstract

Acetamide, is a fundamental structural motif widely utilized in medicinal chemistry and drug design. Its unique combination of chemical stability, hydrogen bonding acceptor and donor capacity, and ease of modification makes it a valuable functional group in the development of therapeutic agents. This review explores the role of acetamide group in enhancing drug-like properties, including solubility, membrane permeability, and metabolic stability. Acetamide moieties are frequently found in a variety of pharmacologically active compounds, such as enzyme inhibitors, anticancer agents, and anti-inflammatory drugs, where they contribute to crucial interactions at the biological target site. Additionally, the incorporation of acetamido structures into lead compounds has been shown to improve binding affinity and pharmacokinetic profiles. Recent advances in synthetic methodologies have further facilitated the incorporation of acetamido group in drug design. In this article we mainly focus on synthesis, biological activity and mechanism of action of acetamido containing FDA approved drugs. This article aims to highlight the versatility and continued relevance of acetamido as a key functional group in the discovery and optimization of modern pharmaceuticals.

## Graphical Abstract

**Figure:**
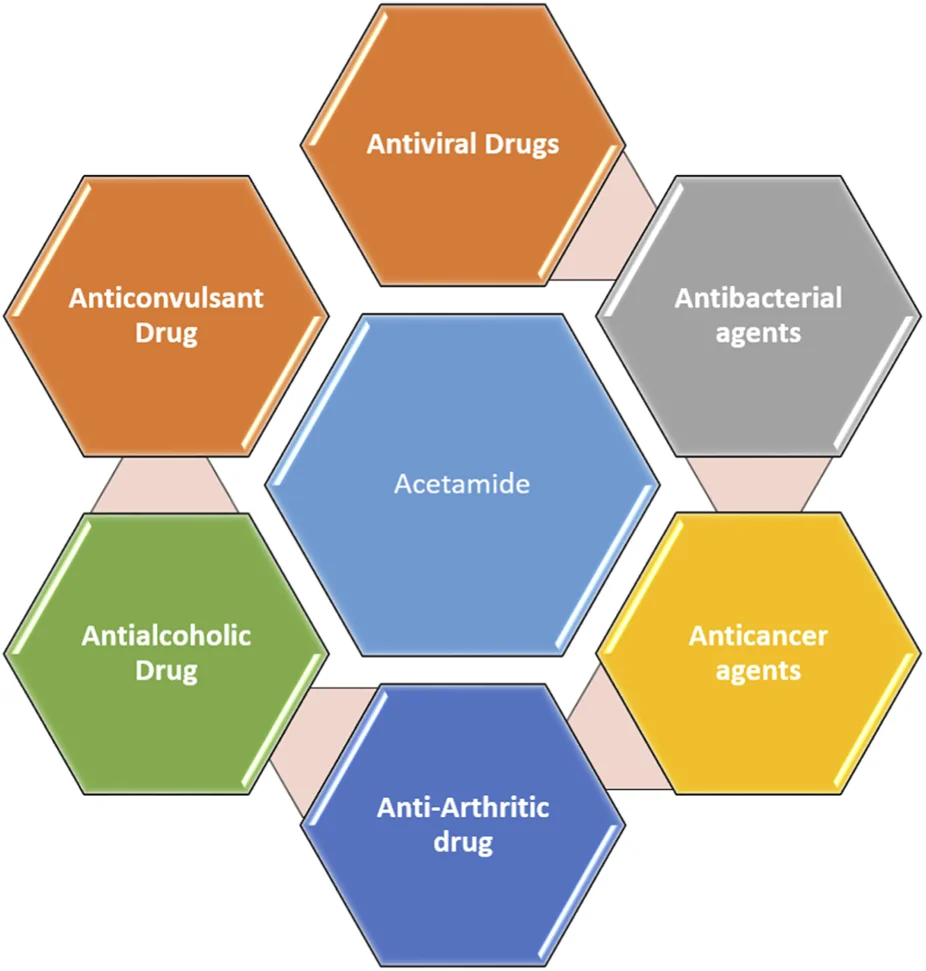

## 1 Introduction

Acetamide, also known as ethanamide (CH_3_CONH_2_), is one of the simplest and most studied members of the acyl amine family. It consists of an acetyl group (CH_3_CO–) linked to an amino group (–NH_2_), forming a primary amide (Srinivasan et al., 2020). Due to its simple structure and fundamental nature, acetamide serves as a model compound for understanding amide chemistry and reactivity. This compound holds significant relevance in both biological and industrial contexts. In biological systems, acetylation is a critical post-translational modification affecting proteins, DNA, and small molecules. Acetamide derivatives are found in numerous biomolecules and pharmaceuticals, playing a role in regulating gene expression, enzyme activity, and metabolic pathways. Industrially, acetamide is used as a plasticizer, solvent, and intermediate in the synthesis of pharmaceuticals, pesticides, and dyes (Kennedy, 2005). The acetamide group is a key structural motif in medicinal chemistry, contributing significantly to both physicochemical and pharmacodynamic properties of drug molecules. Physicochemically, it enhances aqueous solubility through its polar amide bond, while the methyl substitution confers moderate lipophilicity, facilitating passive membrane permeability and, in some cases, blood-brain barrier penetration. The group can engage in both hydrogen bond donation (*via*–NH) and acceptance (*via*–C=O), allowing for favorable interactions with solvent molecules and biological targets. Additionally, the partial double bond character of the amide restricts rotation, imparting conformational rigidity that supports stable binding orientations in target protein sites. Pharmacodynamically, the acetamide moiety contributes to high-affinity binding with enzymes and receptors by forming directional hydrogen bonds within active sites. It also improves metabolic stability compared to esters or other labile functionalities, though it may be susceptible to enzymatic hydrolysis under certain conditions. Overall, the incorporation of an acetamide group can enhance drug-like properties, including bioavailability, target selectivity, and pharmacokinetic performance. Its physicochemical properties, such as its ability to form hydrogen bonds and its relatively high melting point, make it useful in various applications, including as a stabilizer or modifier in polymeric materials. With a wide range of applications, molten acetamide is a good solvent. It can dissolve inorganic compounds with solubilities that are quite similar to those of water because, notably, its dielectric constant is larger than that of the majority of organic solvents (Stafford, 1933). Substitution of acetamide moiety markedly influences their chemical and biological profiles. Compared to free amines, acetamide derivatives exhibit reduced basicity and nucleophilicity, enhanced chemical stability, and improved membrane permeability. These changes often lead to better pharmacokinetic properties, decreased toxicity, and increased selectivity, making N-acylation a valuable strategy in drug design and prodrug development. Acetamide has uses in electrochemistry and the organic synthesis of pharmaceuticals, pesticides, and antioxidants for plastics (Wagner, 2000). Recent research has focused on its role in green chemistry, especially in the development of sustainable amide bond formation strategies, which are crucial for peptide synthesis and drug development. As a building block, acetamide also serves as a starting point for the preparation of more complex nitrogen-containing compounds (Kennedy, 2005). In drug development, the incorporation of acetamido units into small molecules has been shown to enhance pharmacokinetic properties such as solubility, bioavailability, and resistance to enzymatic degradation (Bindra et al., 2024). Acetamide structures are present in a variety of clinically approved drugs, including analgesics, anti-inflammatories, and antineoplastics, underscoring their functional importance (Rani et al., 2014). Advances in synthetic methodologies have further expanded the utility of acetamides, enabling their incorporation under mild, selective, and environmentally friendly conditions. This review explores the chemical characteristics, biological significance, and practical applications of acetamide in drug design, offering insight into its enduring relevance in the pursuit of novel and effective therapeutic agents.

### 1.1 General synthesis of acetamide

#### 1.1.1 Laboratory scale

**SCHEME 1:**
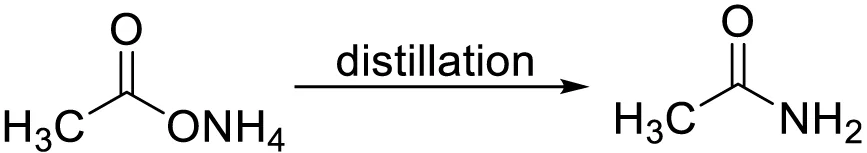
Synthesis of acetamide from ammonium acetate.

**SCHEME 2:**
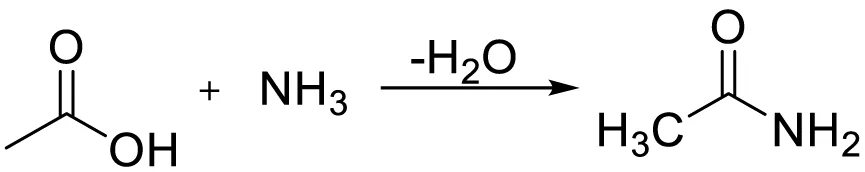
Synthesis of acetamide from acetic acid.

**SCHEME 3:**
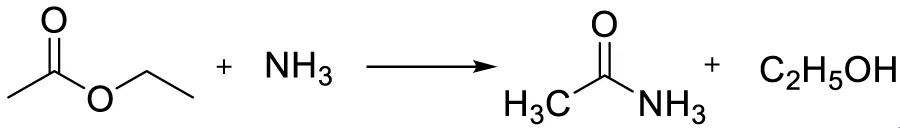
Synthesis of acetamide from ethyl acetate.

Acetamide was prepared in the laboratory by the dehydration of ammonium acetate (Scheme 1) (ACETAMIDE, 1923). Acetamide was synthesized by reacting acetic acid with ammonia (Scheme 2). It was also synthesized by reacting ethyl acetate with ammonia (Scheme 3) (ACETAMIDE, 1923; Al-Ostoot et al., 2021).

#### 1.1.2 Industrial scale

**SCHEME 4:**
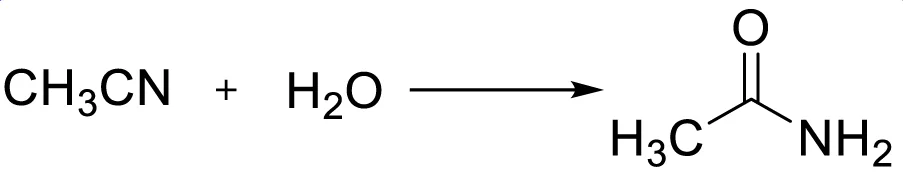
Synthesis of acetamide from acetonitrile.

Similar to certain laboratory techniques, acetamide is made by hydrating acetonitrile, a byproduct of acrylonitrile production, or by dehydrating ammonium acetate (Scheme 4 **) (** Cheung et al., 2011).

## 2 FDA approved drugs containing acetamido groups

### 2.1 Antiviral drugs

#### 2.1.1 Zanamivir

**SCHEME 5:**
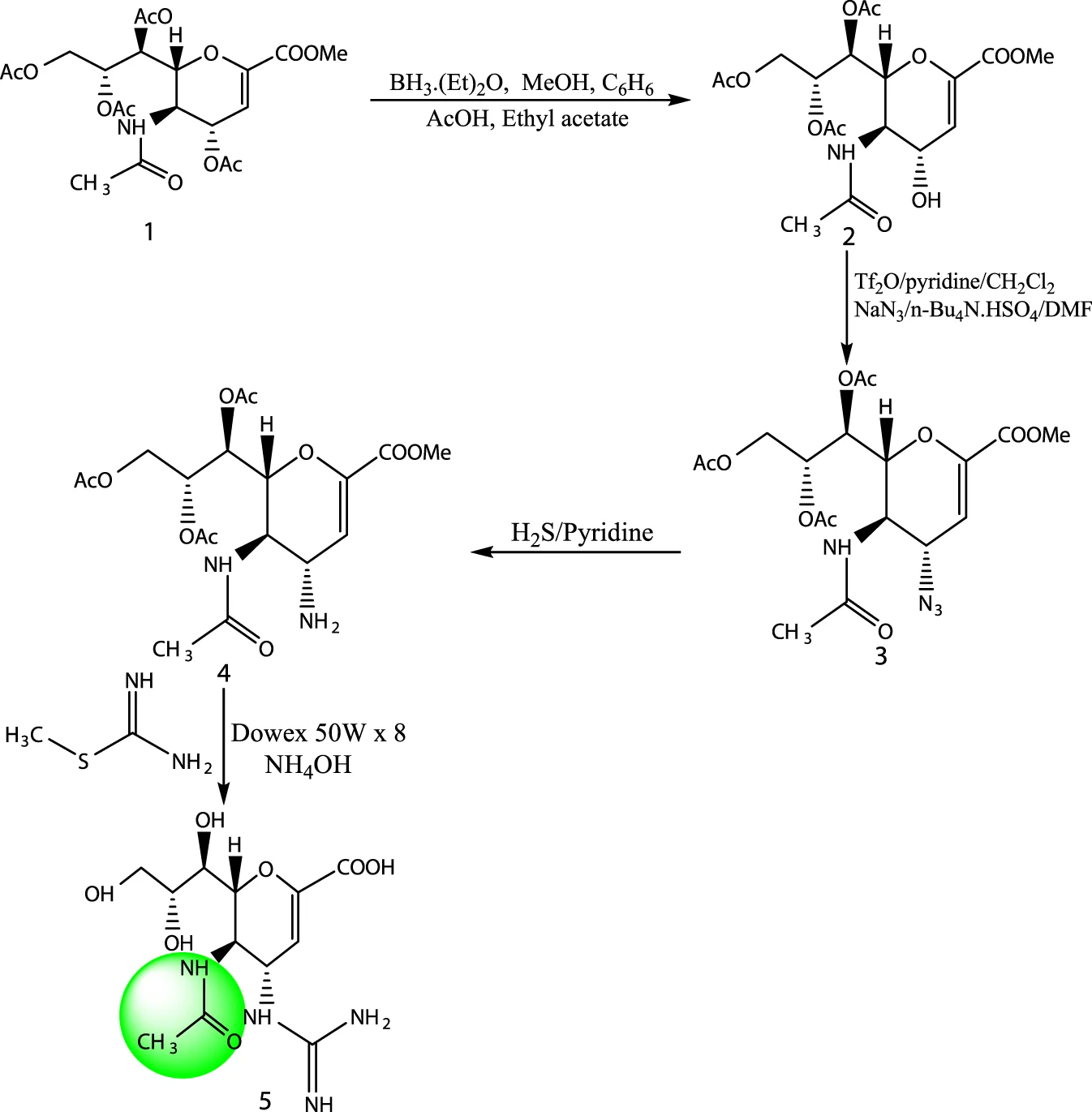
Synthesis of Zanamivir.

Zanamivir is a sialic acid–based neuraminidase inhibitor approved for the treatment of both influenza A and influenza B infections. It was originally developed at Monash University for the treatment and prevention of influenza A and B (Heneghan et al., 2014). Zanamivir is marketed under the brand name Relenza. It was approved in the European Union for the treatment of influenza A and B virus infections through the mutual recognition procedure, following its initial regulatory approval in Sweden in February 1999. It subsequently received approval from the U.S. Food and Drug Administration on 26 July 1999. The acetamide moiety in Zanamivir contributes significantly to neuraminidase inhibition by facilitating critical hydrogen-bonding interactions, mimicking the binding characteristics of sialic acid, and optimizing molecular orientation within the active site, thereby enhancing binding affinity and selectivity toward the viral enzyme (Dunn and Goa, 1999). It is chemically known as (2R,3R,4S)-3-acetamido-4-guanidino-2-((1R,2R)-1,2,3-trihydroxypropyl)-3,4-dihydro-2H-pyran-6-carboxylic acid. It was synthesized by reacting (1S,2R)-1-((2R,3R,4S)-3-acetamido-4-acetoxy-6-(methoxycarbonyl)-3,4-dihydro-2H-pyran-2-yl)propane-1,2,3-triyl triacetate **1** with BH_3_.(Et)_2_O in MeOH and benzene to give compound **2**, which on reaction with Tf_2_O and NaN_3_ in presence of pyridine in dichloromethane to give compound **3**. Compound **3** reacted with H_2_S in presence of pyridine to give compound **4**. The target compound **5** was obtained by reacting compound **4** with methyl carbamimidothioate in presence of ammonium hydroxide with 76% yield (Scheme 5) (Shie et al., 2011).

#### 2.1.2 Oseltamivir

**SCHEME 6:**
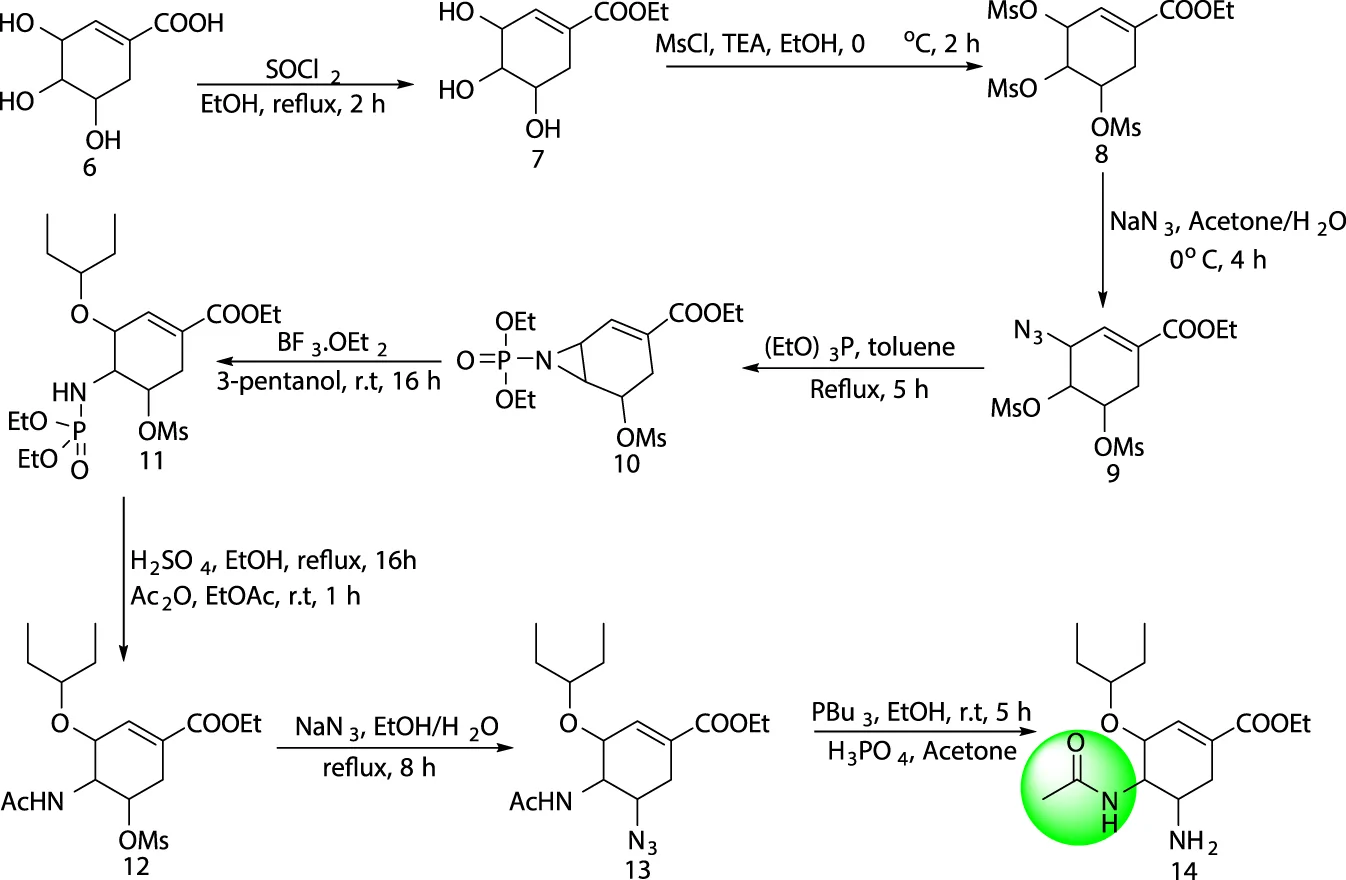
Synthesis of Oseltamivir.

Oseltamivir is another sialic acid analogue based antiviral drug and available as prodrug that undergoes hydrolysis after absorption, converting into its active carboxylic acid form, which strongly inhibits influenza neuraminidases (Tagarro et al., 2019). Oseltamivir is marketed under the brand name Tamiflu and was approved by the U.S. Food and Drug Administration in 1999. The acetamide moiety in Oseltamivir plays a crucial role in neuraminidase inhibition by facilitating key hydrogen-bonding interactions, mimicking the binding behavior of sialic acid, and maintaining an optimal balance between polarity and lipophilicity, thereby enhancing both target binding affinity and oral bioavailability (Tao et al., 2022). It is chemically known as ethyl (3R,4R,5S)-4-acetamido-5-amino-3-(pentan-3-yloxy)cyclohex-1-ene-1-carboxylate. It was synthesized by esterification of 3,4,5-trihydroxycyclohex-1-ene-1-carboxylic acid **6** in the presence of SOCl_2_ and ethanol under reflux condition to give compound **7**, which underwent etherification with MsCl in the presence of TEA in ethanol to give compound **8**. Compound **8** was reacted with Sodium azide in acetone/water mixture to give compound **9**, which was reacted with Triethyl phosphite in toluene under reflux condition to give compound **10**. Compound **10** was reacted with BF_3_. OEt_2_ in 3-pentanol to give compound **11**, which was reacted with H_2_SO_4_ and Ac_2_O in ethanol to give compound **12**. Compound **12** was reacted with sodium azide in ethanol/water mixture to give compound **13**, which was reacted with PBu_3_ in ethanol followed by reacting with H_3_PO_4_ in acetone to give target compound **14** (Oseltamivir) with 70% yield (Scheme 6) (Ishikawa et al., 2009; Rohloff et al., 1998).

#### 2.1.3 Peramivir

**SCHEME 7:**
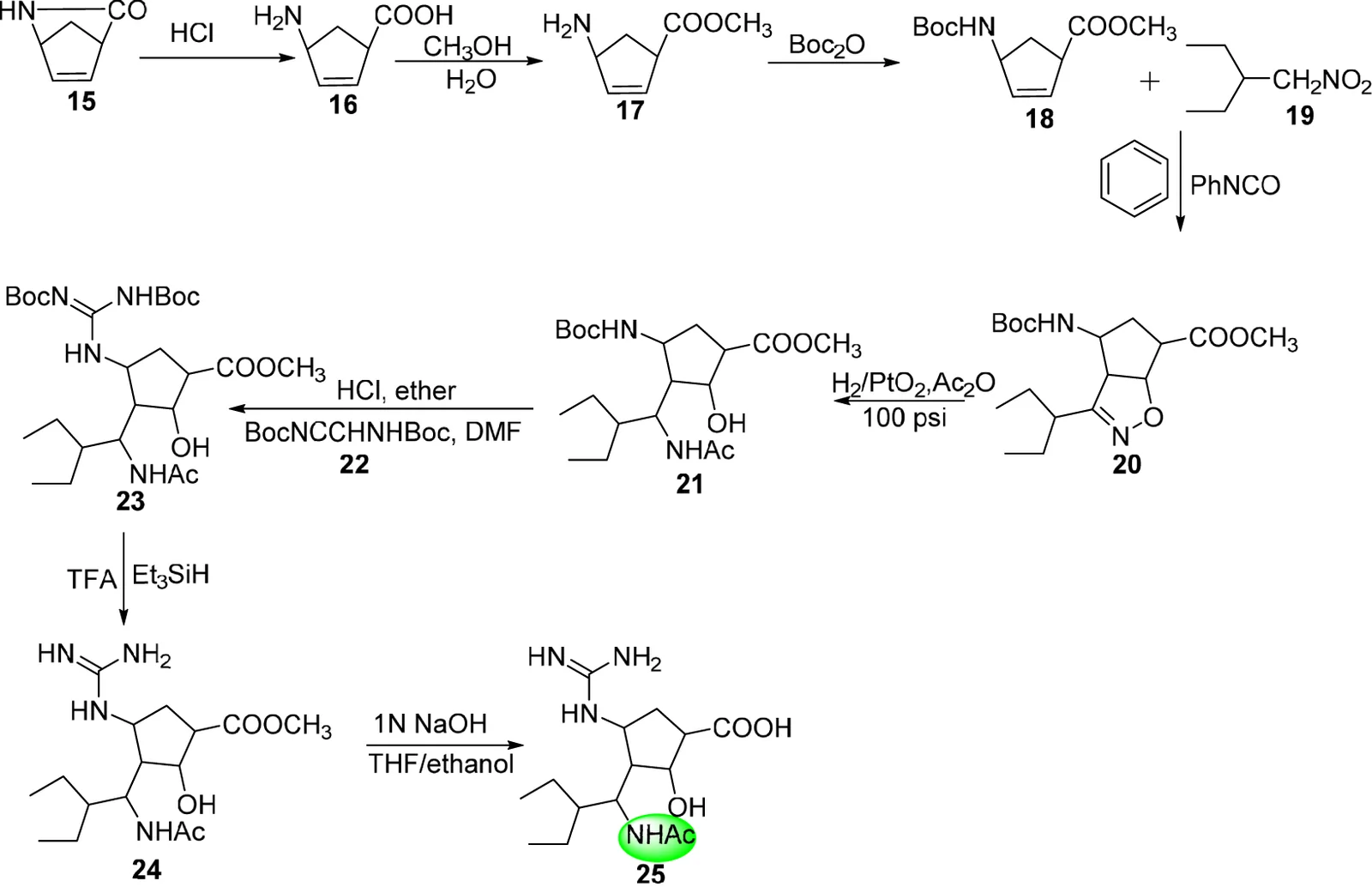
Synthesis of Peramivir.

Peramivir is an antiviral medication used for the treatment of influenza virus infections. It is generally administered by intravenous injection and is particularly useful in patients who are unable to take oral antiviral agents or when rapid therapeutic intervention is required. Peramivir exerts its antiviral activity through inhibition of viral neuraminidase. In addition, it has demonstrated potent activity against multiple influenza strains and, in several studies, has shown greater antiviral potency than Zanamivir and Oseltamivir (Mancuso et al., 2010). The acetamide moiety in Peramivir enhances neuraminidase inhibition by enabling strong hydrogen bonding, mimicking sialic acid interactions, and reinforcing binding affinity while contributing to its polar, intravenous (IV)-compatible profile (Alame et al., 2016). Peramivir was approved by the U.S. Food and Drug Administration on 19 December 2014, and is marketed under the brand name Rapivab. Chemically, Peramivir is known as (1S,2S,3R,4R)-3-((S)-1-acetamido-2-ethylbutyl)-4-guanidino-2-hydroxycyclopentane-1-carboxylic acid. It was synthesized by acidic hydrolysis of the amide bond of 2-azabicyclo[2.2.1]hept-5-en-3-one **15** in the presence of hydrochloric acid to give compound **16**, which underwent esterification with methanol in water to give compound **17**. The amine group of compound **17** was protected with di-tert-butyl dicarbonate to give compound **18**, which was reacted with compound **19** in the presence of isocyanatobenzene to give compound **20**. Compound **20** underwent reduction followed by reaction with acetic anhydride to give compound **21**, which was reacted with hydrochloric acid in ether, followed by reaction with compound **22** in DMF to give compound **23**. The compound **23** was reacted with TFA in triethylsilane to give compound **24**, which was reacted with sodium hydroxide in THF and ethanol to give target compound **25** (Scheme 7) (Smee and Sidwell, 2002).

### 2.2 Antibacterial agents

#### 2.2.1 Linezolid

**SCHEME 8:**
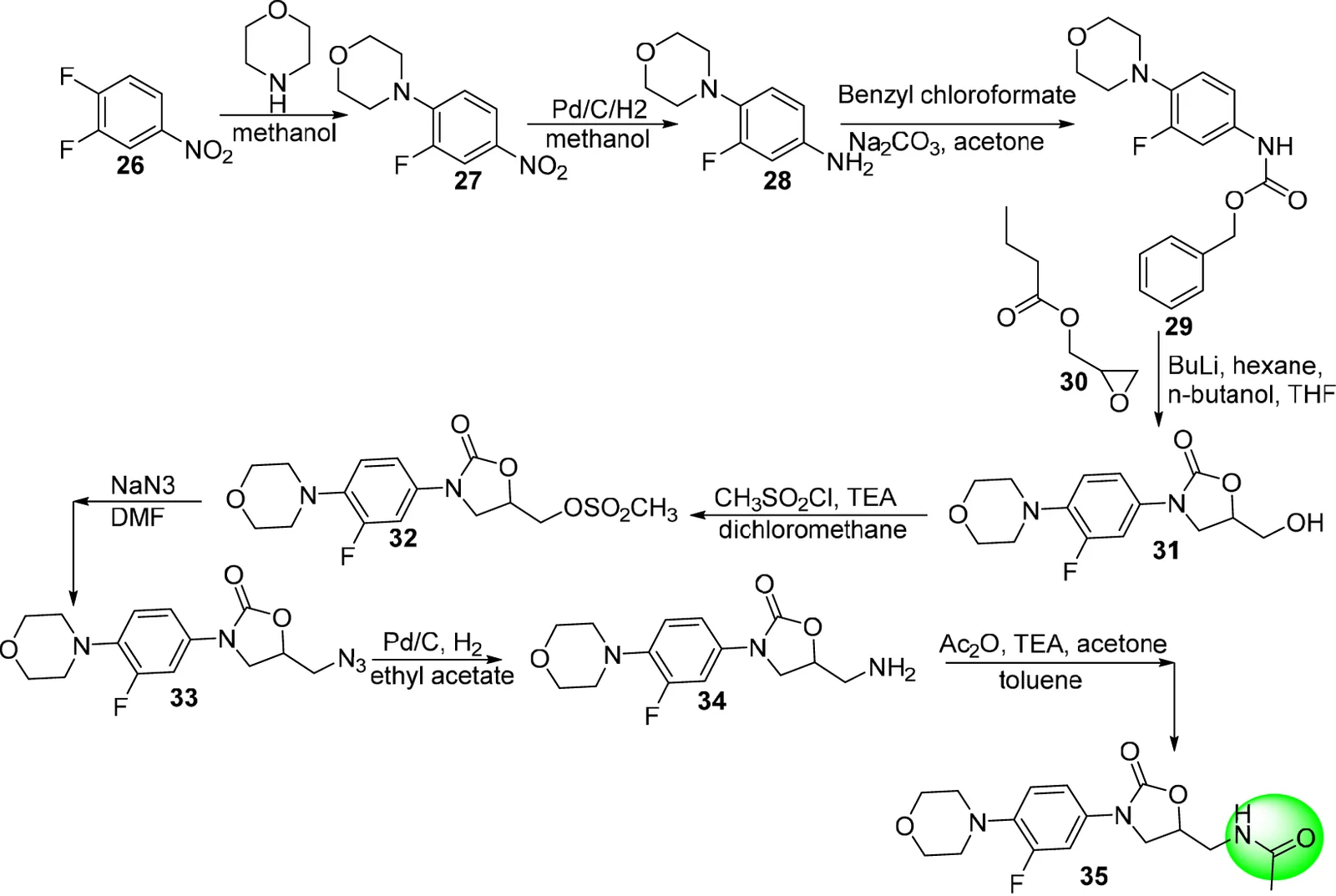
Synthesis of Linezolid.

Linezolid is the first clinically approved drug of the oxazolidinone class of antibacterial agents. It is indicated for the treatment of nosocomial pneumonia and uncomplicated and complicated skin and soft tissue infections caused by Gram-positive bacteria. The acetamide moiety in Linezolid contributes significantly to its antibacterial activity by facilitating key hydrogen-bonding interactions with 23S rRNA, enhancing binding affinity and target selectivity, and maintaining an optimal balance of polarity that supports effective oral bioavailability (Hashemian et al., 2018). Linezolid was discovered by researchers at the Upjohn Company in Kalamazoo (Elbarbry and Moshirian, 2023). It was approved on 18 April 2000. It was sold under the brand name ZYVOX. The acetamide moiety in linezolid plays a crucial role in enhancing metabolic stability by reducing susceptibility to oxidative deamination and enzymatic hydrolysis. Its presence contributes to a balanced physicochemical profile, limiting rapid clearance and supporting the drug’s moderate half-life (Hashemian et al., 2018). It is chemically known as (S)-N-((3-(3-fluoro-4-morpholinophenyl)-2-oxooxazolidin-5-yl)methyl)acetamide. It was synthesized by reacting 1,2-difluoro-4-nitrobenzene **26** with morpholine in methanol to compound **27**, which underwent reduction to give compound **28**. Compound **28** was reacted with benzoyl chloroformate in the presence of sodium carbonate in acetone to give compound **29**, which was reacted with compound **30** in the presence of BuLi in hexane to give compound **31**. Compound **31** was reacted with methanesulfonyl chloride in the presence of TEA in DCM to give compound **32**, which was reacted with NaN_3_ in DMF to give compound **33**. Compound **34** underwent reduction in the presence of Pd/C in ethyl acetate to give compound **34**, which was reacted with acetic anhydride in the presence of TEA in acetone to give target compound **35** with 73% yield (Scheme 8) (Seku et al., 2017).

#### 2.2.2 Sulphacetamide

**SCHEME 9:**
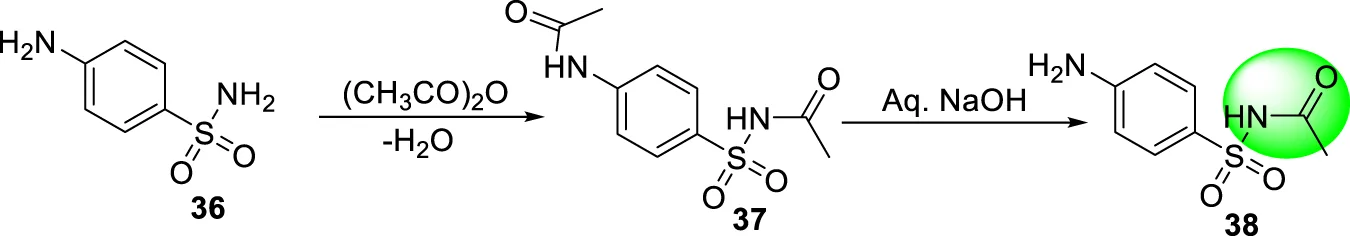
Syntheses of sulfacetamide.

Sulphacetamide is an antibiotic primarily used to treat bacterial infections, particularly those affecting the eyes and skin (Marchand and Nadeau, 1976). It belongs to the class of sulfonamides, which work by inhibiting the growth of bacteria. The acetamide moiety in Sulfacetamide modulates electron density to enhance PABA mimicry, supports hydrogen bonding with DHPS, and improves solubility and tolerability for effective antibacterial activity (Rajesh Goud et al., 2014). Sulfacetamide was first approved by the U.S. Food and Drug Administration (FDA) in 1941**.** It is sold under the brand names Klaron and Ovace. It was synthesized by reacting 4-aminobenzenesulfonamide **36** with acetic anhydride compound **37**, which underwent basic hydrolysis, which selectively removes one acetyl group at *the p*-amine position of compound **37**, resulting in target compound **38** with 80% yield (Scheme 9) (Vardanyan and Hruby, 2006).

#### 2.2.3 Acetohydroxamic acid

**SCHEME 10:**
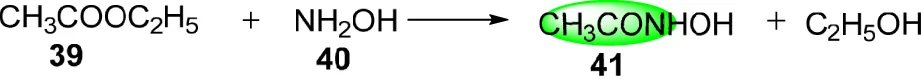
Synthesis of Acetohydroxamic acid.

Acetohydroxamic acid (AHA), also known by the trade name Lithostat, is a powerful and irreversible inhibitor of the urease enzyme found in various bacteria and plants. It is primarily used to treat urinary tract infections (Smolkin et al., 2024). In acetohydroxamic acid, the acetamide-derived hydroxamate moiety enables potent urease inhibition by chelating active-site nickel ions, mimicking the transition state, and stabilizing enzyme binding through hydrogen bonding and electronic effects (Griffith et al., 1978). It was approved by FDA on 31 May 1983. Structurally similar to urea, AHA cannot be hydrolysed by urease, thereby interfering with bacterial metabolism through competitive inhibition. It is chemically known as N-hydroxyacetamide. It was synthesized by reacting ethyl acetate **39** with hydroxylamine **40** to give the target compound **41** (Scheme 10) (Pandey et al., 2011).

### 2.3 Anticancer agents

#### 2.3.1 Abarelix

**SCHEME 11:**
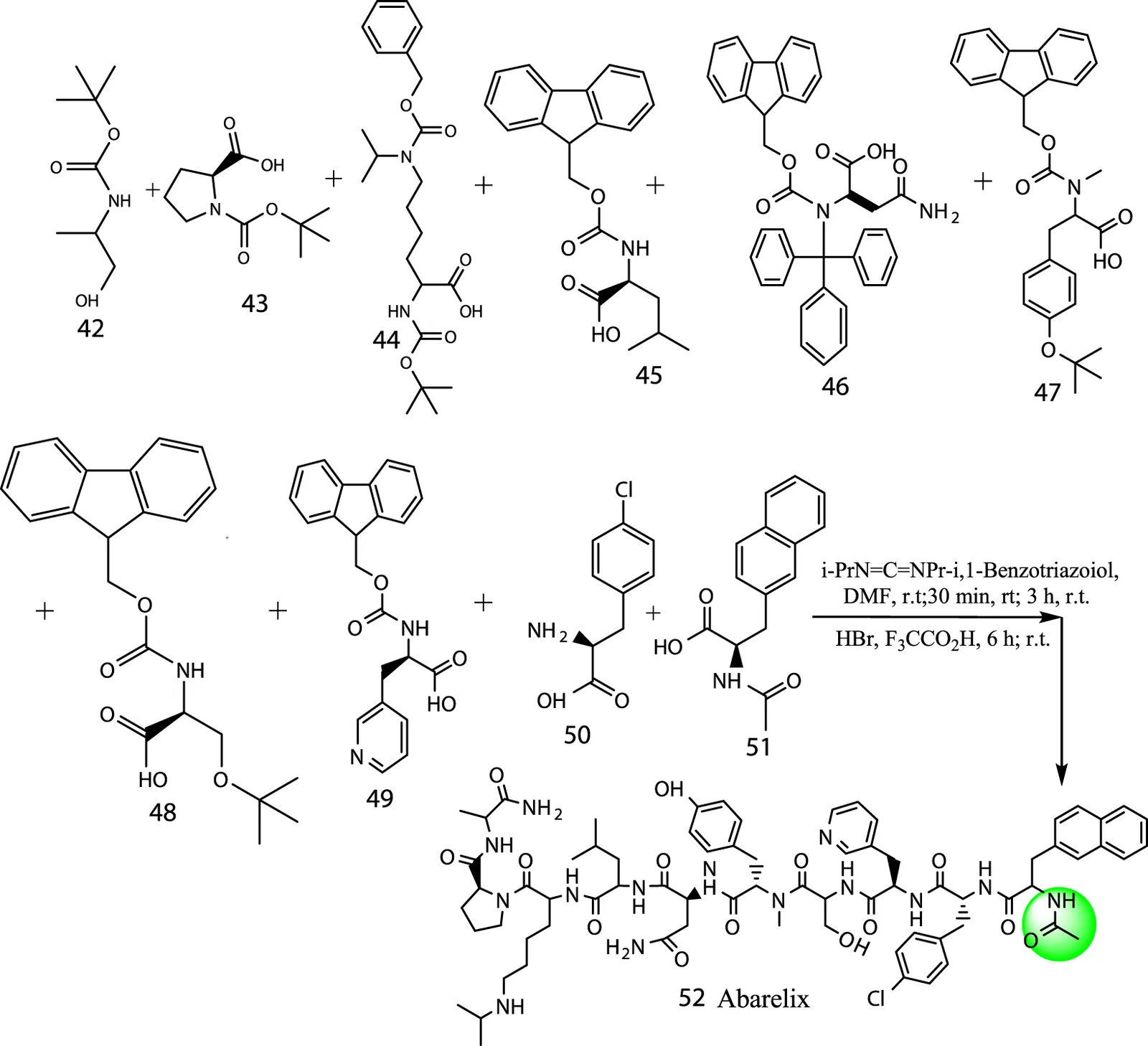
Synthesis of abarelix.

Abarelix is used for the palliative treatment of advanced symptomatic prostate cancer. It is a synthetic decapeptide that acts as an antagonist to gonadotropin-releasing hormone (GnRH) (Kirby et al., 2009). It directly and competitively binds to GnRH receptors in the anterior pituitary gland, blocking their activity and thereby suppressing the secretion of luteinizing hormone (LH) and follicle-stimulating hormone (FSH). In males, this inhibition of LH secretion reduces testosterone production. Consequently, this reduction in testosterone may help alleviate symptoms associated with prostate hypertrophy or prostate cancer, as testosterone is essential for maintaining prostate growth. The acetamide moiety in Abarelix enhances GnRH receptor antagonism by enabling hydrogen bonding, stabilizing peptide conformation, improving solubility, and increasing metabolic stability through amine acetylation (Debruyne et al., 2006). It was approved by the U.S. FDA on 25 November 2003. It is sold under the brand name Plenaxis. It was synthesized by reacting compounds **42–51** together in the presence of 1-benzotriazoiol, hydrogen bromide and trifluoroacetic acid in DMF to give target compound **52** (Scheme 11) (Graul et al., 1998).

#### 2.3.2 Degarelix

**SCHEME 12:**
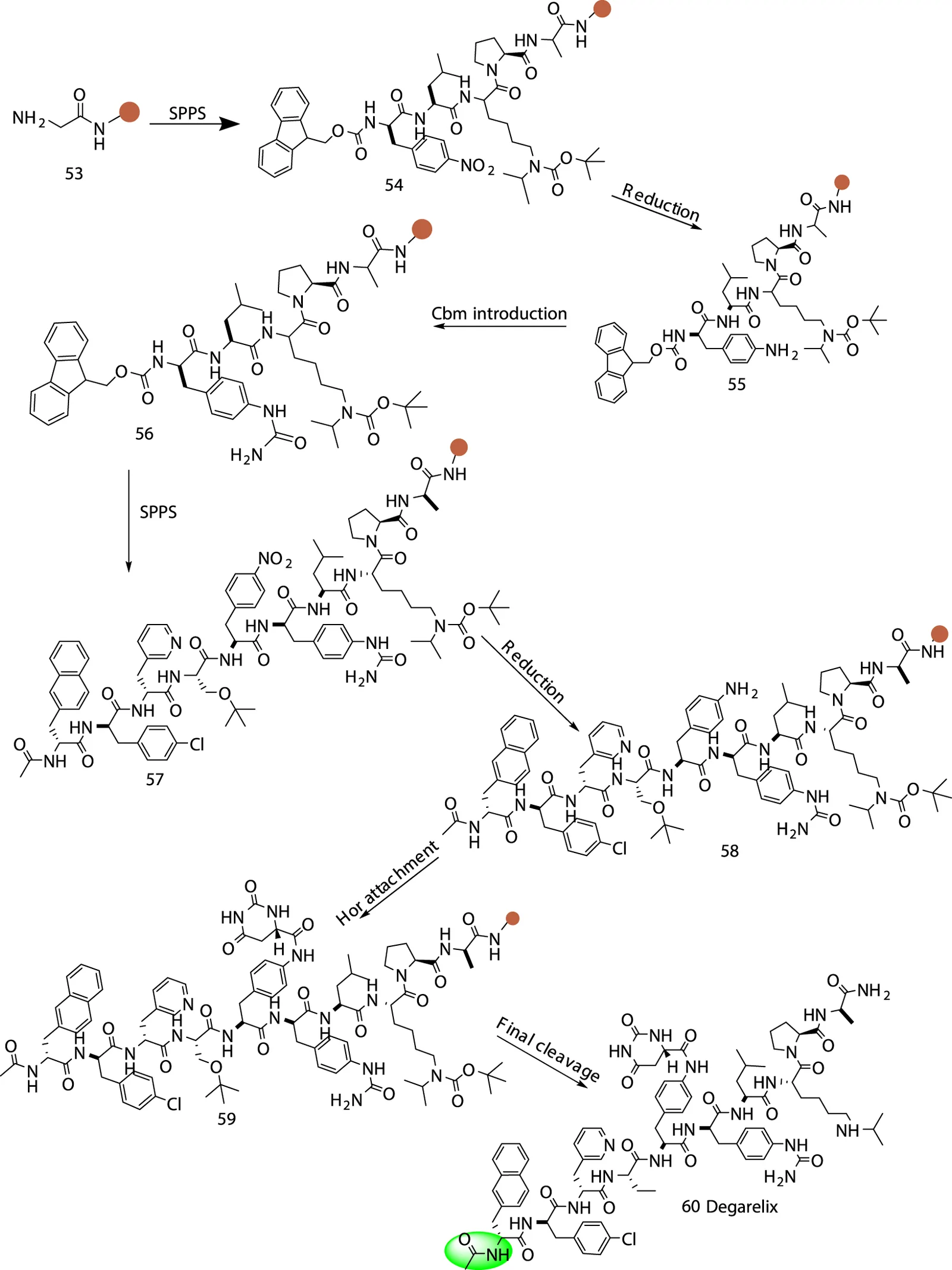
Synthesis of degarelix.

Degarelix is a medication used to treat advanced prostate cancer. It is a gonadotropin-releasing hormone (GnRH) antagonist**,** which works by directly blocking the GnRH receptors in the pituitary gland (Steinberg, 2009). This leads to a rapid decrease in the production of testosterone, a hormone that can fuel the growth of prostate cancer. The acetamide moiety in Degarelix enhances GnRH antagonism by stabilizing peptide conformation, enabling hydrogen bonding, improving metabolic stability, and optimizing polarity for sustained parenteral activity (Ferrazzano et al., 2023). Degarelix was approved by U.S. Food and Drug Administration (USFDA) in December 2008. It is sold under the brand name firmagon. It was synthesized by solid phase synthesis, starting from compound **53** to give compound **54**, which was undergo reduction to give compound **55**. The aminocarbonyl (Cmb) introduction of compound **55** led to formation of compound **56**, which underwent SPPS to give compound **57**. Compound **57** underwent reduction to give compound **58,** which was undergo dihydroorotic (Hor) attachment to give compound **59**. The cleavage of compound **59** result in the formation of target compound **60** with 55% yield (Scheme 12) (Guryanov et al., 2019)**.**

#### 2.3.3 Trametinib

**SCHEME 13:**
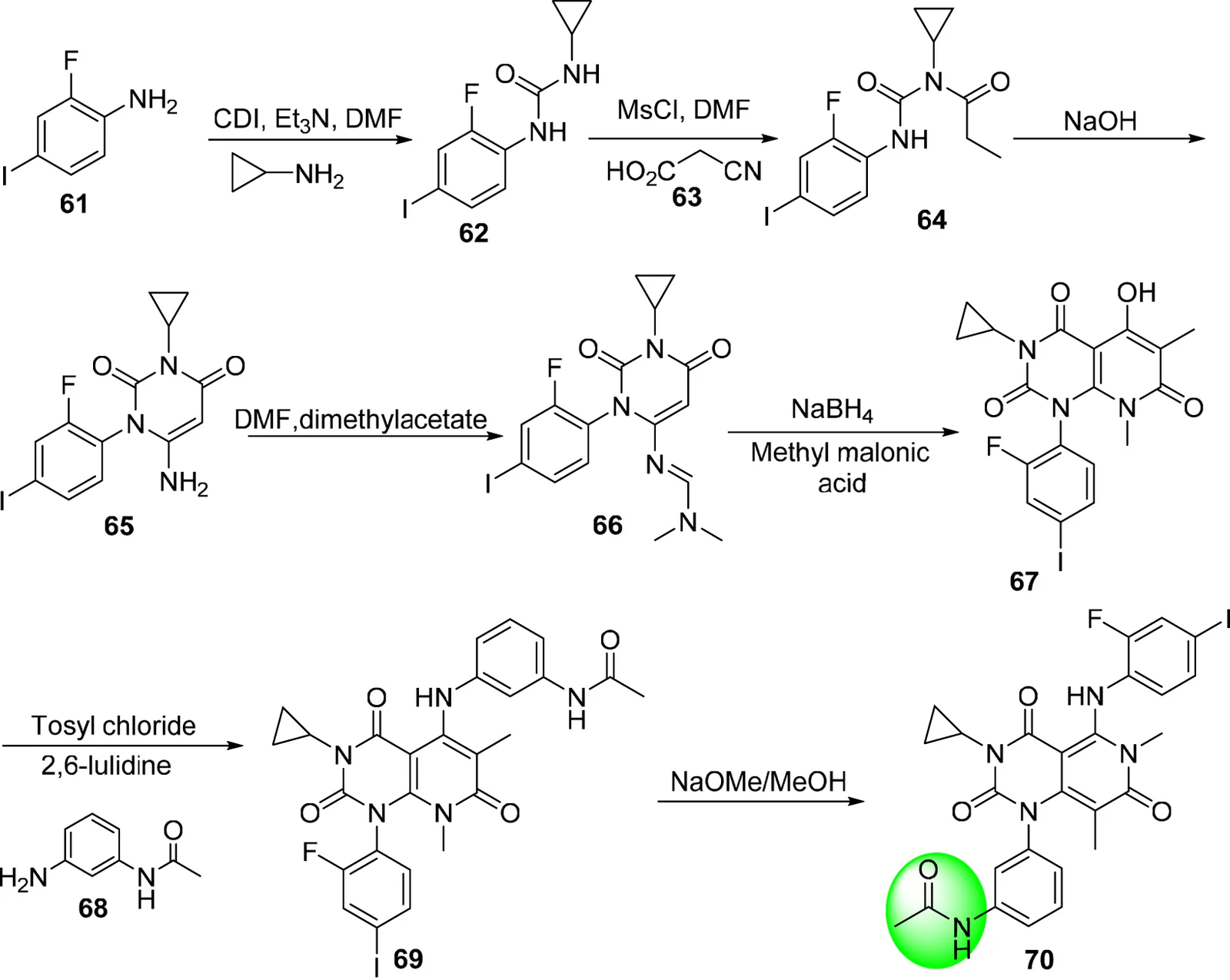
Synthesis of Trametinib.

Trametinib is a medication classified as a MEK inhibitor, used primarily in the treatment of certain types of cancer. Trametinib works by inhibiting MEK1 and MEK2, enzymes in the MAPK pathway (RAS-RAF-MEK-ERK signaling cascade) (Zeiser et al., 2018). This pathway is frequently overactive in cancers with BRAF mutations, promoting cell proliferation and survival. By blocking MEK, Trametinib slows or stops tumor growth. The acetamide moiety in Trametinib enhances MEK inhibition by enabling critical hydrogen bonding at the allosteric site, optimizing molecular orientation, and balancing polarity for improved selectivity and pharmacokinetic performance (Rutkowski et al., 2015). Trametinib exhibits a Cmax of ∼22–30 ng/mL, an oral bioavailability (F%) of ∼70–80%, and a prolonged elimination half-life (t½) of approximately 4–5 days following oral dosing (Leonowens et al., 2014). Trametinib was approved on 29 May 2013. It is sold under the brand name Mekinist. It is chemically known as N-(3-(3-cyclopropyl-5-((2-fluoro-4-iodophenyl)amino)-6,8-dimethyl-2,4,7-trioxo-3,4,6,7-tetrahydropyrido[4,3-d]pyrimidin-1(2H)-yl)phenyl)acetamide. It was synthesized by reacting 2-fluoro-4-iodoaniline **61** with cyclopropylamine in presence of CDI, triethylamine and DMF to give compound **62**, which was reacted with compound **63** in presence of Mesityl chloride in DMF to give compound **64**. Compound **64** underwent cyclisation in presence of sodium hydroxide to give compound **65,** which was reacted with dimethyl acetate in DMF to give compound **66**. Compound **66** underwent cyclisation in presence of NaBH_4_ and methyl malonic acid to give compound **67**, which was reacted with compound **68** in presence of tosyl chloride and 2,6-lulidine to give compound **69**. Compound **69** was reacted with sodium methoxide in methanol to give target compound **70** with 45% yield (Scheme 13) (Millet et al., 2017).

### 2.4 Drug used in treatment of hyperparathyroidism

#### 2.4.1 Etelcalcetide

**SCHEME 14:**
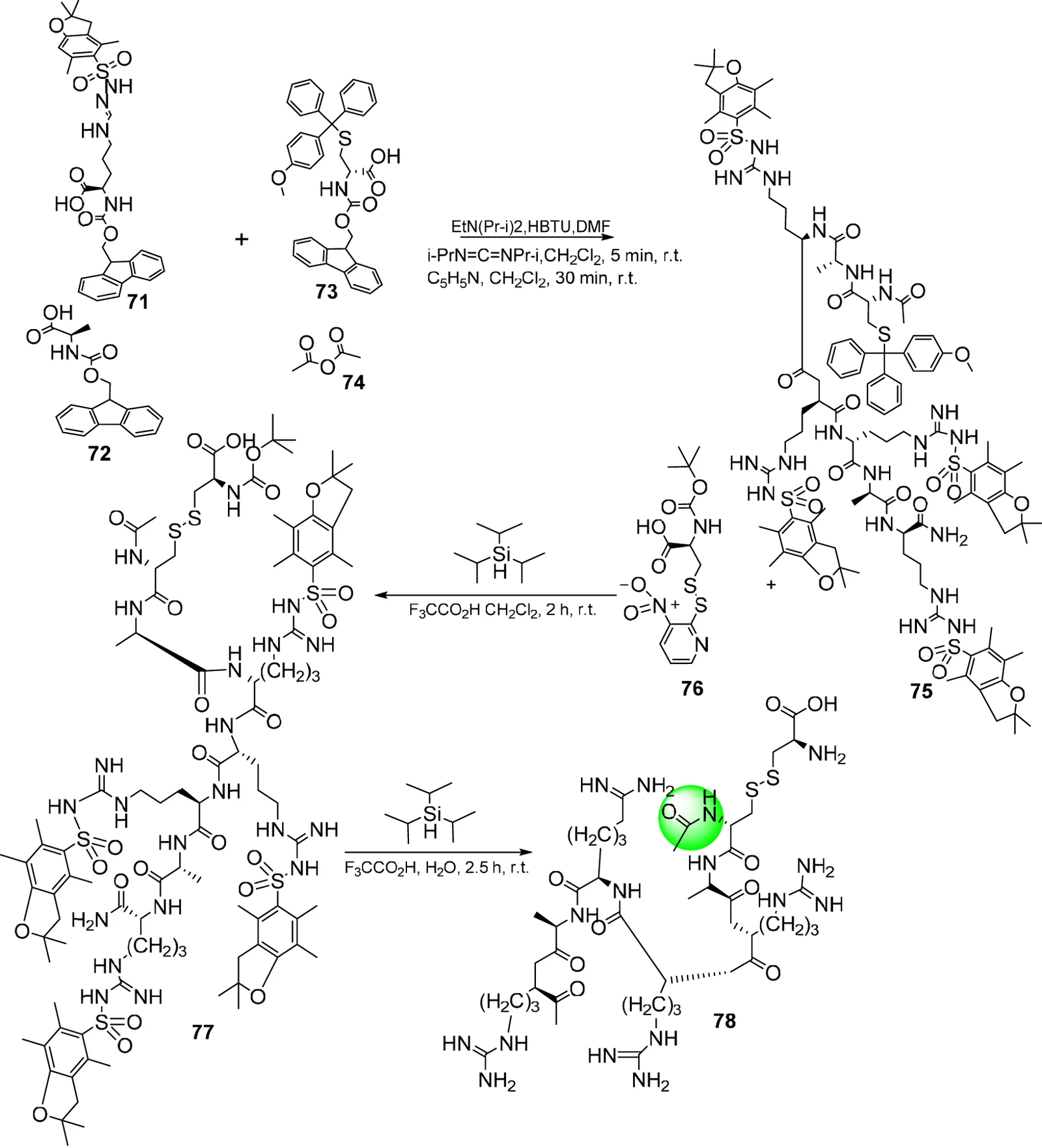
Synthesis of Etelcalcetide.

Etelcalcetide hydrochloride is a novel peptide calcimimetic agent that has a similar mechanism of action as cinacalcet hydrochloride. Etelcalcetide hydrochloride (AMG 416/ONO-5163, formerly velcalcetide, KAI-4169) is a novel calcimimetic agent that was developed for the treatment of hyperparathyroidism (SHPT) (Hamano et al., 2017). Etelcalcetide is a peptide that consists of 8 amino acids. The acetamide moiety in Etelcalcetide enhances CaSR activation by stabilizing peptide conformation, enabling hydrogen bonding, improving metabolic stability, and optimizing polarity for intravenous therapeutic use (Yu et al., 2017). It was approved by U.S. Food and Drug Administration on 7 February 2017. It is sold under the brand name Parsabiv. It was synthesized by reacting **71–74** together in presence of diisopropylethylamine and HBTU in DMF to give compound **75**, which was reacted with compound **76** in presence of triisopropylsilane and trifluoroacetic acid in dichloromethane to give compound **77**. Compound **77** was reacted with triisopropylsilane in presence of trifluoroacetic acid in water to form target compound **78** (Scheme 14) (Blair, 2016).

### 2.5 Anti-arthritic drug

#### 2.5.1 Apremilast

**SCHEME 15:**
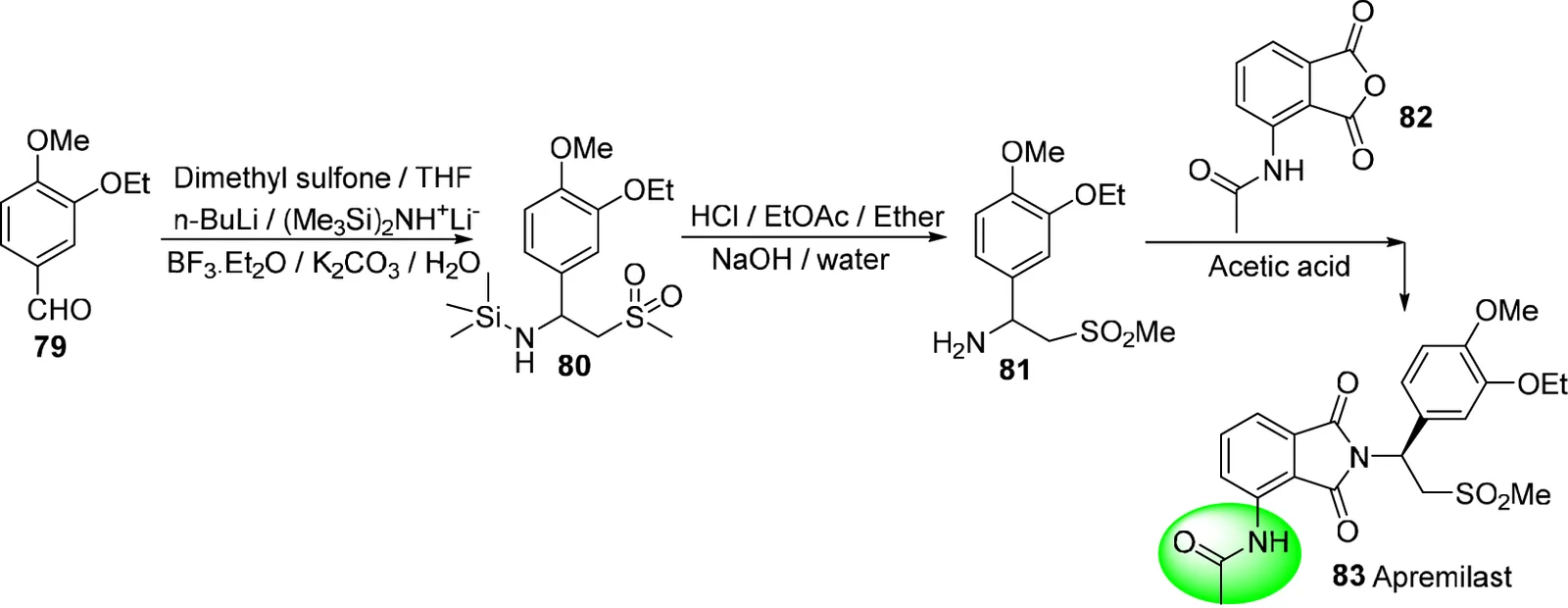
Synthesis of Apremilast.

Apremilast is an orally administered small molecule that specifically inhibits phosphodiesterase-4 (PDE4) enzyme and modulates the immune system by increasing the levels of intracellular cyclic adenosine monophosphate (cAMP), and inhibiting IL-2 and IL-8, interferon-γ, and tumor necrosis factor (TNF) production. It is used for the treatment of psoriasis and psoriatic arthritis (Nassim et al., 2020). The acetamide moiety in Apremilast enhances PDE4 inhibition by enabling key hydrogen bonding, optimizing molecular orientation, and balancing polarity for effective oral bioavailability and selectivity (Schett et al., 2010). It is sold under the brand name Otezla. The FDA approved apremilast on 21 March 2014. It is chemically known as (S)-N-(2-(1-(3-ethoxy-4-methoxyphenyl)-2-(methylsulfonyl)ethyl)-1,3-dioxoisoindolin-4-yl)acetamide. It was synthesized by reacting 3-ethoxy-4-methoxybenzaldehyde **79** with dimethyl sulfone in THF in the presence of n-BuLi followed by the reaction with BF_3_. Et_2_O/K_2_CO_3_ in water to give compound **80**, which was reacted with HCl/EtOAc in ether followed by neutralization with sodium hydroxide to give compound **81**. Compound **81** was reacted with compound **82** in presence of acetic acid to give target compound **83** with 59% yield (Scheme 15) (Narode et al., 2021).

#### 2.5.2 Colchicine

**SCHEME 16:**
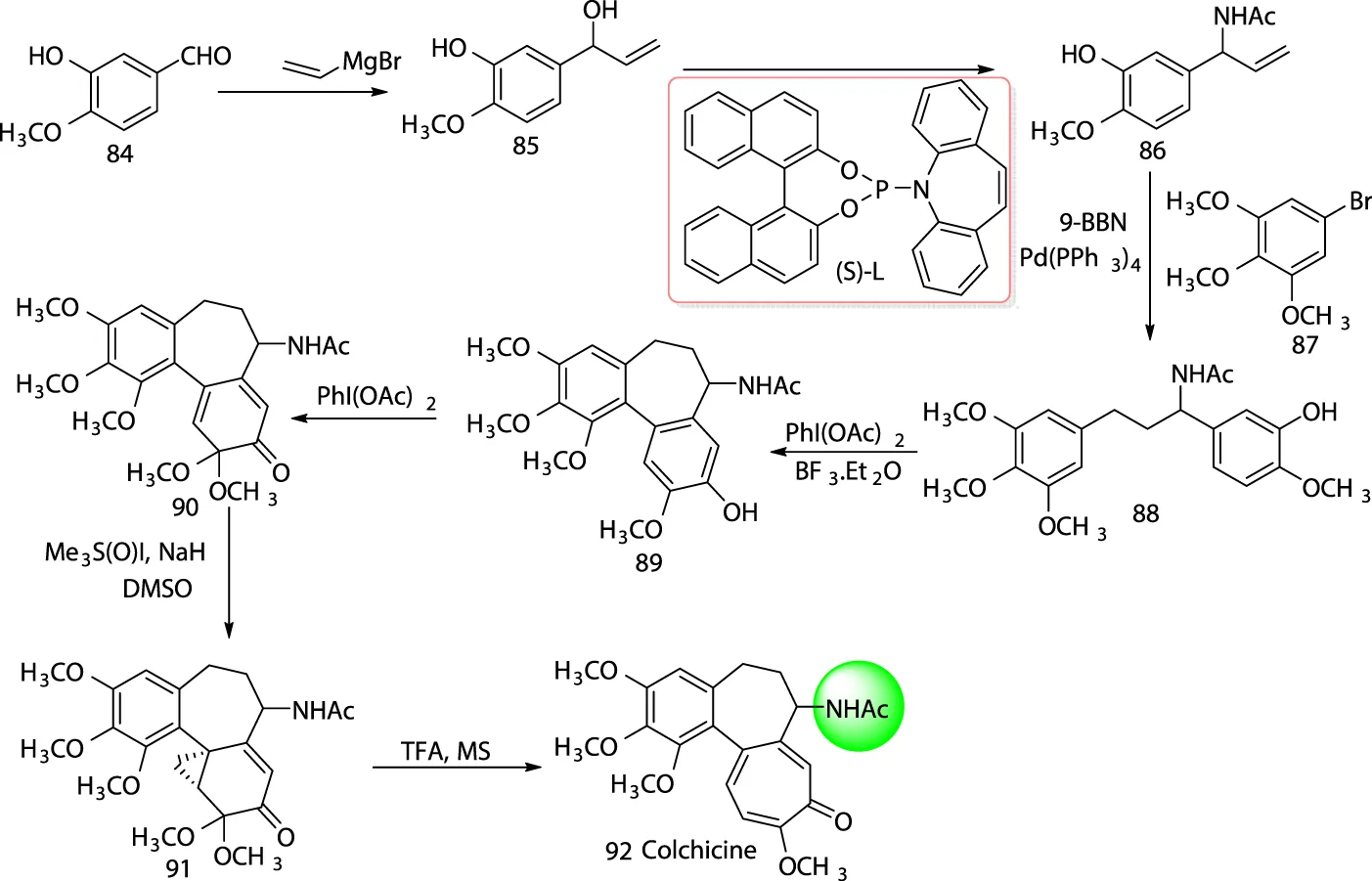
Synthesis of Colchicine.

Colchicine is a tricyclic alkaloid with anti-inflammatory properties, derived from the herbaceous *Colchicum autumnale* plant, and was first isolated and synthesized in the 19th century (Tucker et al., 2024). It is one of the few drugs that have persisted from antiquity to modern times, having been mentioned in a 1550 BC Egyptian papyrus and utilized by ancient Greek, Byzantine, and Arabian physicians. Historically, colchicine has been used to treat gout and, since its discovery in the 1970s, has been employed to prevent attacks of the hereditary autoinflammatory disease familial Mediterranean fever (FMF) and its most severe complication, AA amyloidosis (Pascart and Richette, 2018). The acetamide moiety in Colchicine is crucial for tubulin binding, enabling key hydrogen bonding interactions, maintaining proper molecular orientation, and ensuring high biological activity (Schattner, 2022). It is chemically known as N-(1,2,3,10-tetramethoxy-9-oxo-5,6,7,9-tetrahydrobenzo[a]heptalen-7-yl)acetamide. It was approved by FDA in July 2009 for the treatment of three conditions: familial Mediterranean fever, acute gout flares, and the prevention of gout flares. It was sold under the brand name Colcrys (Scheme 16). It was synthesized by reacting isovanillin **84** with Grignard reagent to give secondary allylic alcohol **85**, which underwent acylation in presence of (S)-L catalyst to give Allylic acetamide **86**. Compound **86** was converted in to alkyl borane by using 9-BBN, which was further reacted with 3,4,5-trimethoxylphenyl bromide **87** in presence Pd(PPh_3_)_4_ catalyst to give biphenyl compound **88.** Compound **88** was reacted with PhI(OAc)_2_ and BF_3_. Et_2_O to give allocolchicinoid **89**, which was reacted with PhI(OAc)_2_ to give compound **90**. Nucleophilic cyclopropanation of compound **90** with dimethylsulfoxonium methylide gives tetracyclic product **91**, which was reacted with TFA in DCM to give Colchicine **92** with 82% yield, along with an unrearranged product caused by the simple hydrolysis of acetal, which was efficiently inhibited using a 4-Å molecular sieve (Scheme 16) (Liang et al., 2021).

### 2.6 GnRH antagonists

#### 2.6.1 Cetrorelix

**SCHEME 17:**
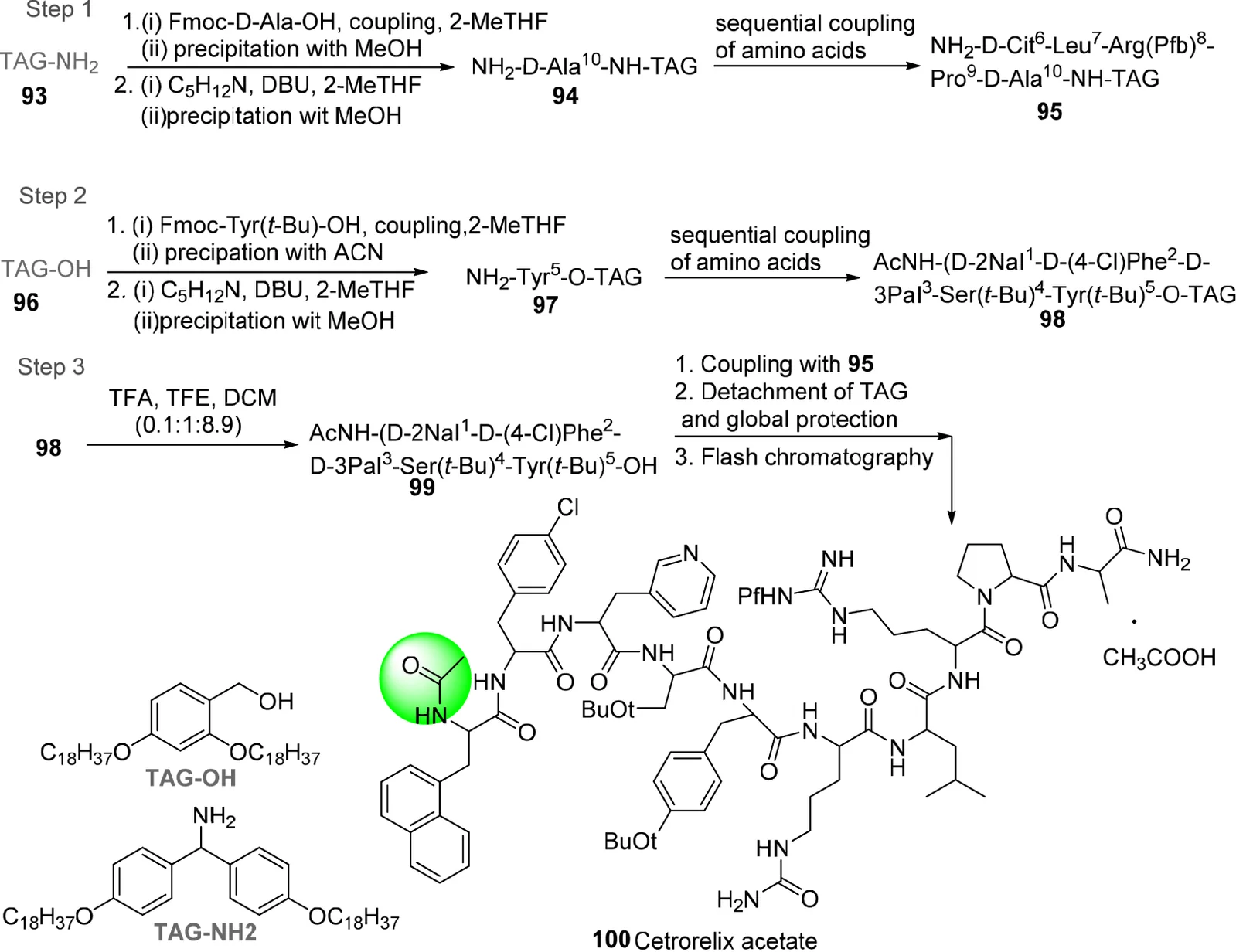
Tag method for the Synthesis of Cetrorelix Acetate.

Cetrorelix is a pharmaceutical compound, specifically a gonadotropin-releasing hormone (GnRH) antagonist. It is often used in clinical and research settings for its ability to regulate reproductive hormones (Finas et al., 2006). GnRH antagonists work by blocking the action of GnRH on the pituitary gland, suppressing the release of luteinizing hormone (LH) and follicle-stimulating hormone (FSH). It is used to prevent premature ovulation as part of controlled ovarian stimulation treatment or in conditions like prostate cancer, endometriosis, or uterine fibroids, where hormonal control is needed (Tur-Kaspa and Ezcurra, 2009). The acetamide moiety in Cetrorelix enhances GnRH antagonism by enabling hydrogen bonding, stabilizing peptide conformation, improving metabolic stability, and optimizing polarity for parenteral administration (Tur-Kaspa and Ezcurra, 2009). It was approved by FDA on 11 August 2000. It is sold under the brand name Cetrotide. It is chemically known as 1-((2-(2-(2-(2-(2-(2-acetamido-3-(naphthalen-2-yl)propanamido)-3-(4-chlorophenyl)propanamido)-3-(pyridin-3-yl)propanamido)-3-hydroxypropanamido)-3-(4-hydroxyphenyl)propanamido)-5-ureidopentanoyl)leucylarginyl)-N-(1-amino-1-oxopropan-2-yl)pyrrolidine-2-carboxamide. The cetrorelix was synthesized by the convergent synthetic method in three steps. In step one, TAG-NH_2_ **93** was subjected to the amino acid coupling followed by precipitation with methanol to give compound **94**, which underwent sequential coupling of amino acids to give compound **95**. In step two, TAG-OH **96** was subjected to the amino acid coupling followed by precipitation with acetonitrile to give compound **97**, which underwent sequential coupling of amino acids to give compound **98**. In step three, the TAG group of compound **98** was detached by using TFA, TEF in DCM to give compound **99**, which was coupled with compound **95**, followed by detachment of TAG and global protection to give cetrorelix **100** with 50% overall yield (Scheme 17) (Avula et al., 2026).

#### 2.6.2 Ganirelix

**SCHEME 18:**
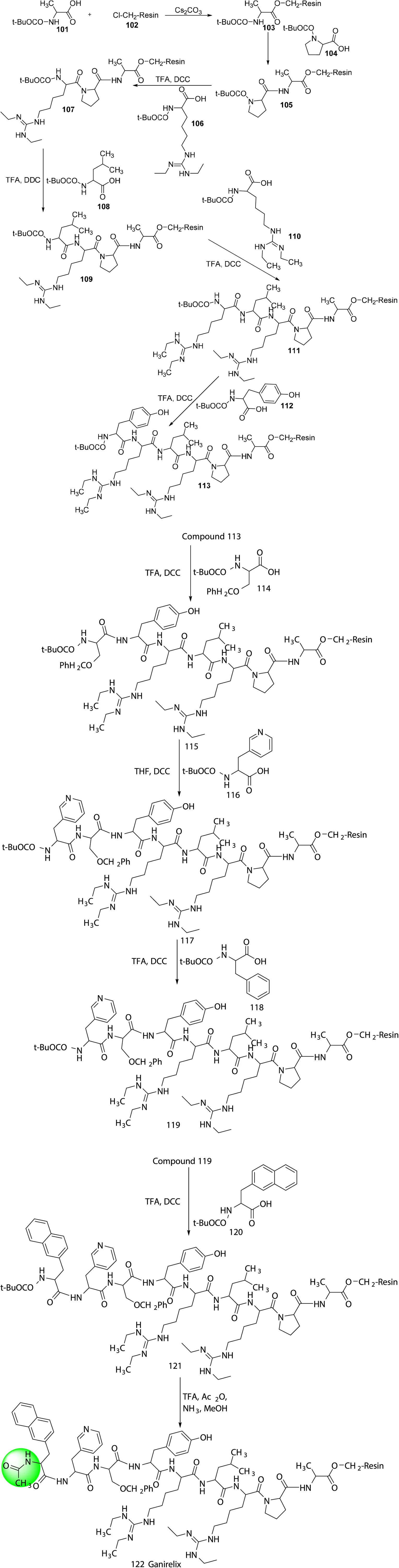
Synthesis of Ganirelix.

Ganirelix is a medication used in fertility treatments to prevent premature ovulation in women undergoing controlled ovarian hyperstimulation (Gillies et al., 2000). It works by inhibiting the action of gonadotropin-releasing hormone (GnRH), thereby preventing the early release of eggs from the ovaries. It is a GnRH antagonist that works by competitively blocking the GnRH receptors in the pituitary gland**.** This prevents the natural release of luteinizing hormone (LH) and follicle-stimulating hormone (FSH), thereby suppressing premature ovulation during controlled ovarian stimulation in fertility treatments (Mannaerts 2023). The acetamide moiety in Ganirelix enhances GnRH antagonism by enabling hydrogen bonding, stabilizing peptide conformation, improving metabolic stability, and optimizing polarity for effective parenteral use (Gillies et al., 2000). It was approved FDA on 29 July 1999. It was sold under the brand name Antagon. It is chemically known as (S)-1-(N^2^-N^2^-((R)-2-((R)-2-((R)-2-acetamido-3-(naphthalen-2-yl)propanamido)-3-(4-chlorophenyl)propanamido)-3-(pyridin-3-yl)propanoyl)-L-seryl-L-tyrosyl-N6-(bis(ethylamino)methylene)-D-lysyl-L-leucyl-N6-(bis(ethylamino)methylene)-L-lysyl)-N-((R)-1-amino-1-oxopropan-2-yl)pyrrolidine-2-carboxamide. It was synthesized by reacting compound **101** with chloromethylresine **102** in presence of cesium carbonate to give compound **103**, which was reacted with compound **104** to form amide derivatives **105**. Compound **105** underwent peptide coupling with the acid group of compound **106** in the presence of coupling agents TFA and DCC to give compound **107**, which was reacted with compound **108** in the presence of TFA and DCC to give compound **109**. Compound **109** underwent peptide coupling with compound **110** in the presence of coupling agent TFA and DCC to give compound **111**, which was reacted with the acid group of compound **112** to form a new amide bond in the presence of TFA and DCC to give compound **113**. The tert-butoxy carbonyl protecting group of compound **113** was reacted with the acid group of compound **114** to form a new amide bond by the peptide coupling method in the presence of coupling agents TFA and DCC to give compound **115**, which was reacted with compound **116** in the presence of TFA and DCC to give compound **117**. Compound **117** underwent the same peptide coupling process with compound **118** to give compound **119**, which further underwent the same peptide coupling with compound **120** in the presence of coupling agents TFA and DCC to give compound **121**. Compound **121** was reacted with TFA, acetic anhydride and ammonia in methanol to give the target compound Ganirelix **122** (Scheme 18) (Chatterjee and Bandyopadhyay, 2023).

### 2.7 Drug used in treatment of obesity

#### 2.7.1 Setmelanotide

**SCHEME 19:**
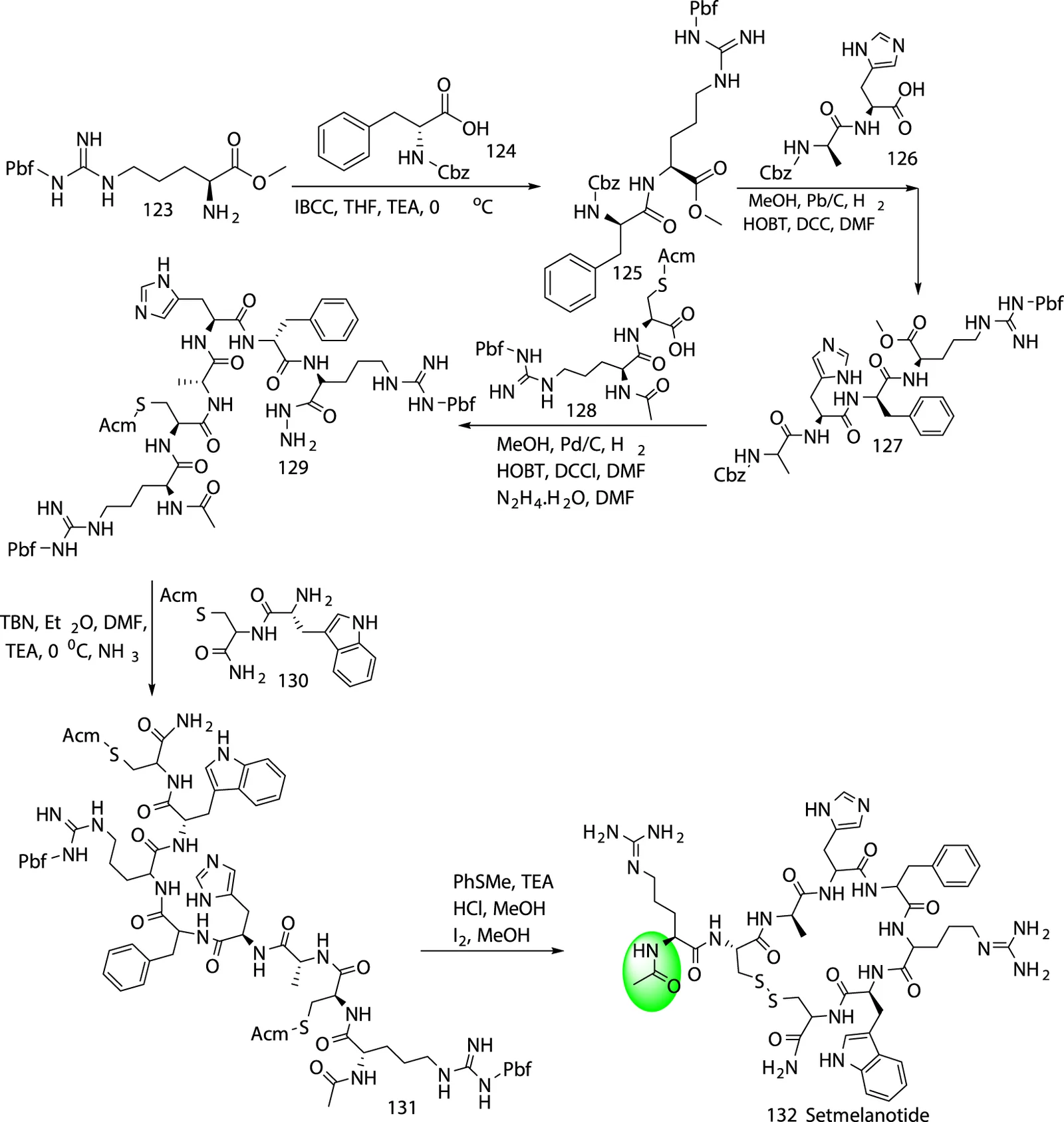
Synthesis of Setmelanotide.

It is a melanocortin-4 receptor (MC4R) agonist used to treat obesity caused by specific genetic disorders affecting the MC4R pathway (Haqq et al., 2022). It activates the MC4 receptor, which plays a key role in appetite regulation and energy balance. Patients with mutations in this pathway often experience extreme hunger (hyperphagia) and early-onset severe obesity by stimulating MC4R. The acetamide moiety in Setmelanotide enhances MC4R agonism by stabilizing peptide conformation, enabling hydrogen bonding, improving metabolic stability, and optimizing polarity for effective receptor interaction (Peng et al., 2026). It was approved by the FDA on 25 November 2020 for weight management in patients with pro-opiomelanocortin (POMC), proprotein convertase subtilisin/kexin type 1 (PCSK1), or leptin receptor (LEPR) deficiency (Markham, 2021). It was sold under the brand name imcivree. It is chemically known as(7S,19R,22R)-16-((1H-imidazol-5-yl)methyl)-7-((1H-indol-3-yl)methyl)-22-((S)-2-acetamido-5-((diaminomethylene)amino)pentanamido)-13-benzyl-10-(3((diaminomethylene)amino)propyl)-19-methyl-6,9,12,15,18,21-hexaoxo-1,2-dithia-5,8,11,14,17,20-hexaazacyclotricosane-4-carboxamide. It was synthesized by reacting H-Arg (Pbf)-OMe **123** with Z-D-Phe-OH **124** in presence of isobutyl chlorocarbonate (IBCC) and THF in TEA to give compound **125**, which was reacted with compound **126** in presence of Pb/C in methanol under hydrogen atmosphere to give compound **127**. Compound **127** was reacted with compound **128** in the presence of Pb/C in methanol under a hydrogen atmosphere, followed by reaction with hydrazine hydrate in DMF to give compound **129**, which was reacted with compound **130** in presence of tert-butyl nitrite (TBN) and diethyl ether in DMF, followed by the reaction with ammonia to give compound **131**. The target compound **132** was obtained by reacting compound **131** with methyl(phenyl)sulfane in the presence of TEA in methanol, followed by reaction with Iodine in methanol (Scheme 19) (Yuan et al., 2021).

### 2.8 Nonsteroidal anti-inflammatory drug (NSAID)

#### 2.8.1 Acetaminophen

**SCHEME 20:**
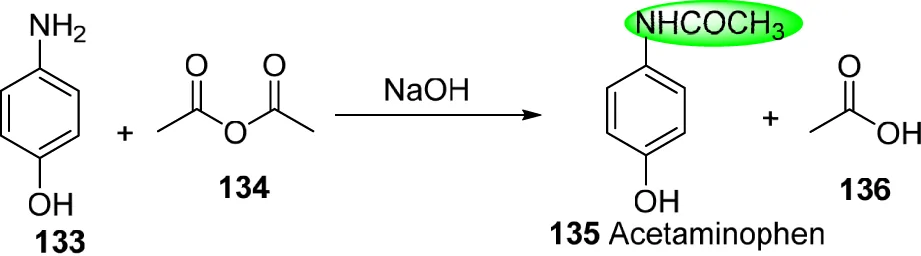
Synthesis of acetaminophen.

Acetaminophen, also known as N-acetyl-*p*-aminophenol (APAP) or paracetamol, is one of the most widely used analgesics (pain reliever) and antipyretics (fever reducer) (Aminoshariae and Khan, 2015). The acetamide moiety in Acetaminophen modulates reactivity and toxicity, enables hydrogen bonding with COX enzymes, and optimizes polarity and metabolism for effective analgesic and antipyretic activity (Kouznetsov, 2024). It was first approved by the FDA in 1951. It was sold under the brand names Tylenol and Panadol. It has been widely used as an active ingredient in many approved drugs. According to the U.S. FDA, there are currently 235 approved prescription and over-the-counter drug products that contain acetaminophen as an active ingredient. Acetaminophen is extremely safe and effective when used as prescribed, but hepatotoxicity and irreversible liver damage can occur when taken excessively or in combination with alcohol. Acetaminophen undergoes extensive phase II metabolism *via* glucuronidation and sulfation, yielding non-toxic conjugates. The acetamide moiety plays a critical role in stabilizing the aromatic system and favouring these detoxification pathways, while limiting excessive oxidative activation. However, a minor fraction is metabolized by cytochrome P450 enzymes to the reactive intermediate N-acetyl-p-benzoquinone imine (NAPQI), which, under conditions of overdose, overwhelms glutathione defences and leads to hepatotoxicity (McGill and Jaeschke, 2013). It is chemically known as N-(4-hydroxyphenyl)acetamide. It was synthesized by reacting *para*-aminophenol **133** with acetic anhydride **134** in the presence of sodium hydroxide to give target compound **135** with 56.5% yield (Scheme 20) (Chiew et al., 2018).

### 2.9 Antialcoholic drug

#### 2.9.1 Acamprosate

**SCHEME 21:**
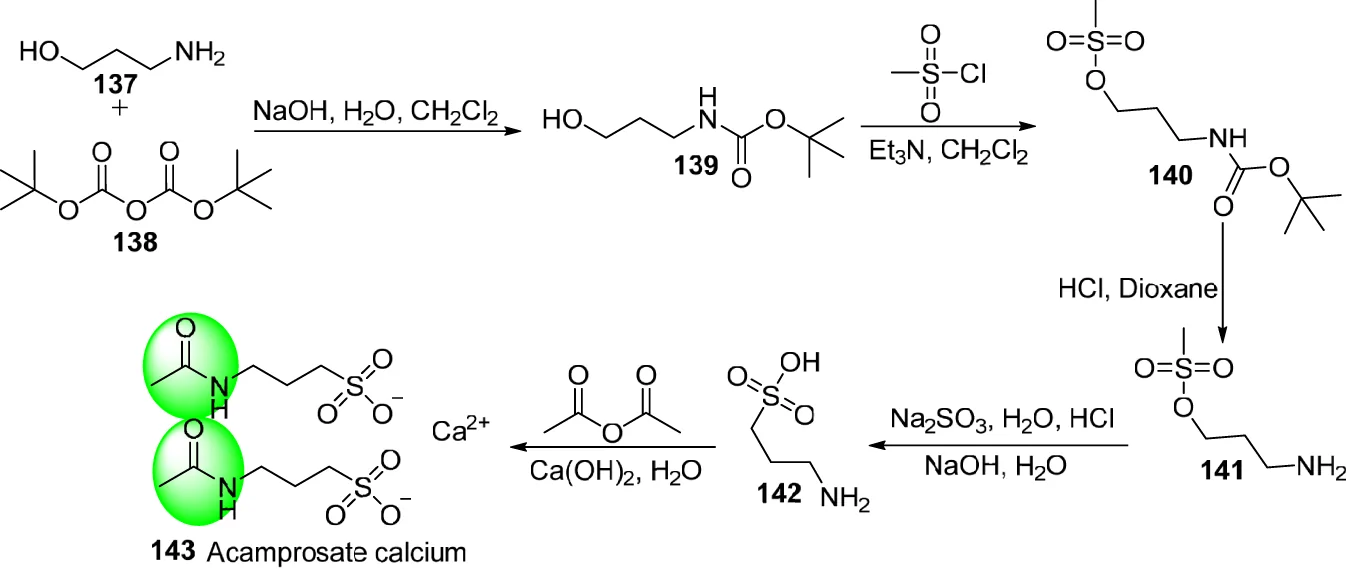
Synthesis of acamprosate calcium.

Acamprosate is a medication used to help people maintain abstinence from alcohol after they have stopped drinking (Kennedy et al., 2010). It is commonly prescribed for individuals with alcohol use disorder (AUD) to reduce cravings and prevent relapse. Acamprosate works by stabilizing the balance of neurotransmitters in the brain, particularly glutamate and GABA, which are disrupted by long-term alcohol use. It modulates glutamatergic neurotransmission to reduce alcohol cravings and withdrawal symptoms. The acetamide moiety in Acamprosate supports neurotransmitter mimicry, enables hydrogen bonding, and contributes to high polarity and stability, facilitating modulation of glutamatergic signaling in alcohol dependence treatment (Lee et al., 2011). It was approved on 29 July 2004. It was sold under the brand name Campral. It was synthesized by reacting 3-aminopropan-1-ol **137** with di-tert-butyl dicarbonate **138** in the presence of sodium hydroxide in dichloromethane to give compound **139**, which was reacted with methanesulphonyl chloride in the presence of triethylamine in dichloromethane to give compound **140**. Compound **140** was reacted with hydrochloric acid in dioxane to give compound **141**, which was reacted with sodium sulphite and sodium hydroxide to give compound **142**. Target compound **143** was obtained by reacting compound **142** with acetic anhydride in the presence of calcium hydroxide in a very good yield 92% (Scheme 21) (Kufahl et al., 2014).

### 2.10 Drug used in treatment of paracetamol overdose

#### 2.10.1 N-Acetylcysteine

**SCHEME 22:**
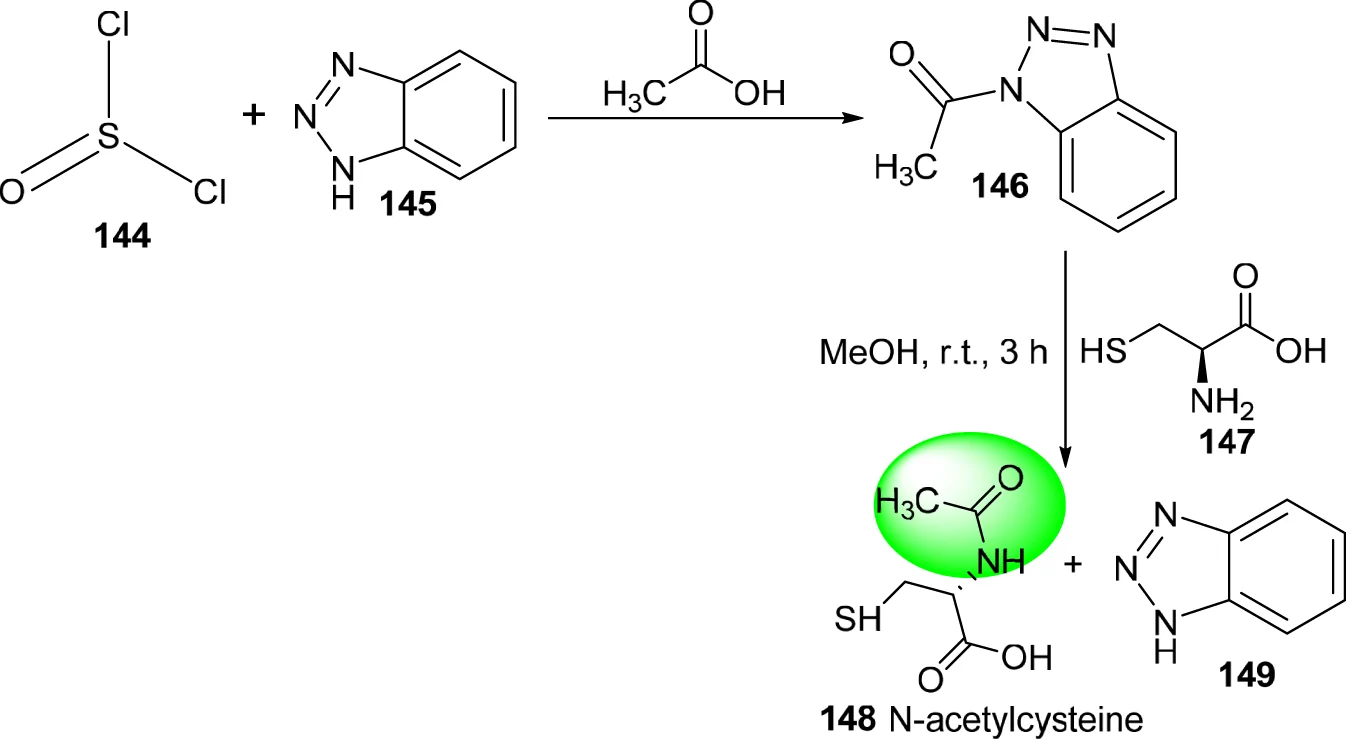
Synthesis of N-acetylcysteine.

N-acetylcysteine is an antioxidant and is used as an antidote to paracetamol (acetaminophen) overdose. N-acetylcysteine can support the body’s antioxidant level during infections, toxic assaults, inflammations, and stresses (Holdiness, 1991). It is often prescribed to patients who are at high risk for hepatotoxicity. N-acetylcysteine is a precursor to make glutathione, which is the major antioxidant in the human body. It was shown that taking N-acetylcysteine as a supplement also increases the levels of glutathione. Glutathione can detoxify many toxic compounds, including peroxide, xenobiotic substances, and other radical species. It might also ameliorate lung inflammation occurring in influenza. The acetamide moiety in N-acetylcysteine enhances stability and tolerability by reducing amine reactivity, while preserving the active thiol group for antioxidant, mucolytic, and detoxifying actions (Raghu et al., 2021). It was approved by the FDA on 14 September 1963. It was sold under the brand names ACC 200, Acetadote, Fluimucil and Mucomyst. It is chemically known as acetyl-L-cysteine. It was synthesized by reacting sulfurous dichloride **144** with 1H-benzo[d][1,2,3]triazole **145** in the presence of glacial acetic acid to give compound **146**, which was reacted with compound **147** in methanol at room temperature to give target compound **148** with 70% yield (Scheme 22) (Ziaee and Ziaee, 2021).

### 2.11 Drug used in treatment of phototoxicity

#### 2.11.1 Afamelanotide

**SCHEME 23:**
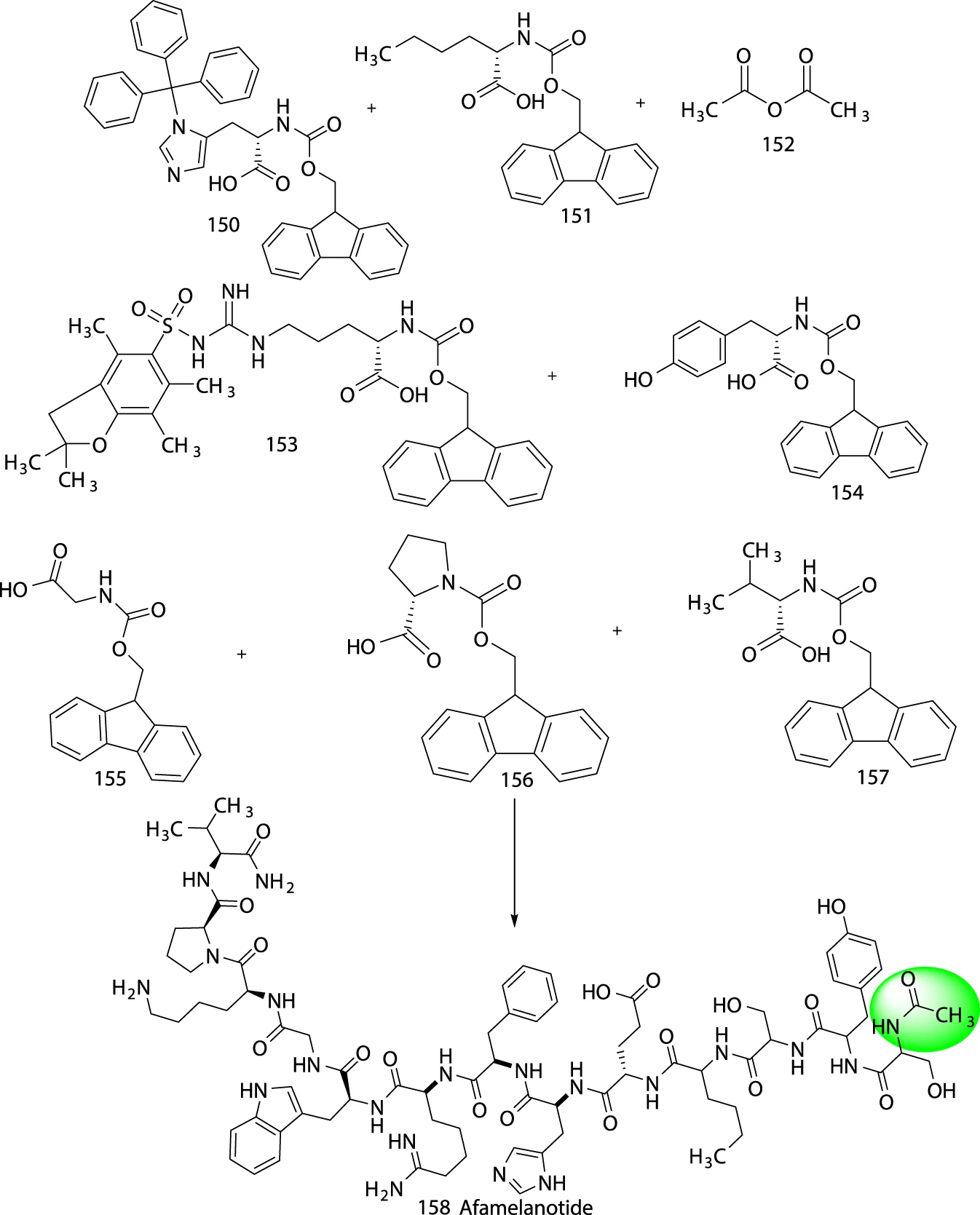
Synthesis of Afamelanotide.

Afamelanotide, the first synthetic analogue of α-MSH, was developed in the 1980s at the University of Arizona. Although synthetic, it mimics the action of endogenous α-MSH by activating the melanocortin-1 receptor (MC1R) involved in eumelanogenesis (Wensink et al., 2021). Structurally, afamelanotide is a synthetic tridecapeptide and functions as a melanocortin receptor agonist, with a higher binding affinity and prolonged receptor interaction compared to natural α-MSH. Its enhanced resistance to rapid degradation by proteolytic enzymes contributes to its extended biological activity. Although afamelanotide is hydrolysed quickly, the pharmacokinetics and pharmacodynamics of its metabolites remain incompletely understood. It is believed to replicate the pharmacological actions of endogenous MSH by promoting eumelanin production *via* MC1R activation. Eumelanin, in turn, offers photoprotective benefits, including broad-spectrum absorption of UV and visible light, antioxidant effects through free radical scavenging, inactivation of superoxide anions, and upregulation of superoxide dismutase to mitigate oxidative stress (Wu and Cotliar, 2021). In animal studies, it demonstrated up to 1,000-fold greater activity, stability, and ability to stimulate tyrosinase. Notably, afamelanotide can induce melanogenesis without UV exposure, though UV light enhances and prolongs the effect by stimulating epidermal proliferation. Moreover, both *in vitro* and *in vivo* studies have shown that afamelanotide may enhance DNA repair in keratinocytes following UV-induced damage. Initial clinical trials focused on patients with erythropoietic protoporphyria (EPP). The acetamide moiety in Afamelanotide enhances melanocortin-1 receptor activation by stabilizing peptide conformation, enabling hydrogen bonding, improving metabolic stability, and optimizing polarity for sustained therapeutic activity (Polańska et al., 2024). It was approved by the U.S FDA on 8 October 2019 for the prevention of Phototoxicity in Erythropoietic Protoporphyria. It is sold under the brand name Scenesse. It was synthesized by reacting compounds **150–157** together to give target compound **158 (** Scheme 23) (Kim and Garnock-Jones, 2016)**.**

### 2.12 Anticonvulsant drug

#### 2.12.1 Lacosamide

**SCHEME 24:**
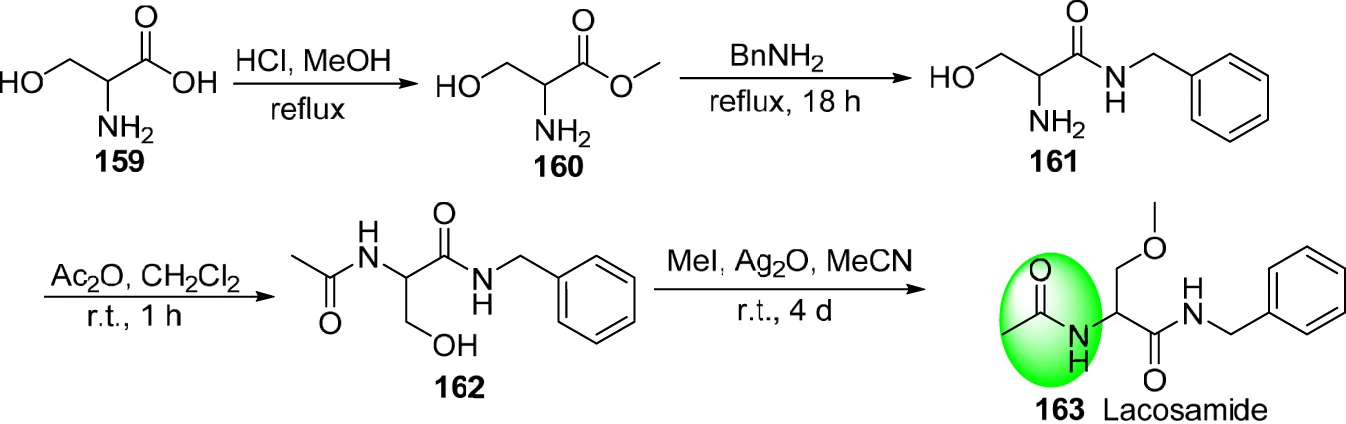
Synthesis of Lacosamide.

Lacosamide is a member of a series of functionalized amino acid molecules that have been screened for anticonvulsant properties (Andurkar et al., 1999). Research has demonstrated that lacosamide is an effective anticonvulsant in animal models (Stöhr et al., 2007). Clinically, lacosamide has been evaluated as a monotherapy for individuals with painful diabetic neuropathy and is currently in the late stages of development as an additional treatment for patients with uncontrolled partial-onset seizures. The ideal lacosamide dosage, according to the results of completed research, is between 200 and 600 mg per day (Doty et al., 2007). The acetamide moiety in Lacosamide supports selective enhancement of sodium channel slow inactivation by enabling hydrogen bonding, stabilizing molecular conformation, and optimizing polarity and metabolic stability (Errington et al., 2008). It was first approved by the FDA on 28 October 2008 for the treatment of partial-onset seizures in adults. It was sold under the brand name Vimpat. It is chemically known as 2-acetamido-N-benzyl-3-methoxypropanamide. It was synthesized by esterification of serine **159** with methanol in the presence of hydrochloric acid to give compound **160**, which was reacted with benzylamine to give compound **161**. Compound **161** was reacted with acetic anhydride in dichloromethane to give compound **162**, which was reacted with methyl iodide in the presence of silver oxide and methyl cyanide to give target compound **163** with 82% yield (Scheme 24) (Yang et al., 2019).

### 2.13 Diuretics

#### 2.13.1 Acetazolamide

**SCHEME 25:**
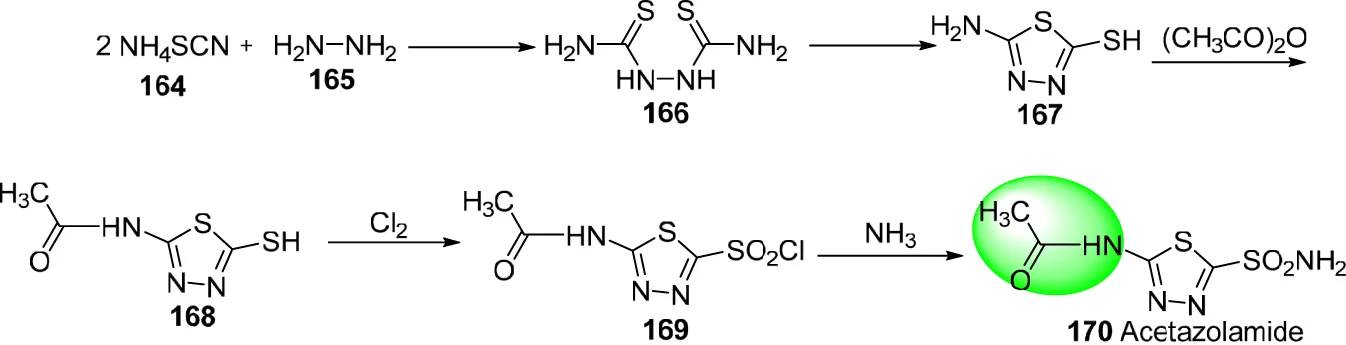
Synthesis of Acetazolamide.

Acetazolamide is a sulphonamide-based carbonic anhydrase inhibitor. Acetazolamide is primarily used as a medication to reduce elevated intraocular pressure associated with glaucoma. Nevertheless, it is a diuretic that raises urine pH by increasing the excretion of bicarbonate. These characteristics may offer some clinical advantages for conditions including high altitude erythropoiesis, metabolic alkalosis, nephrolithiasis, rhabdomyolysis, contrast-induced nephropathy (CIN), and sleep apnoea. It can be an effective adjunct treatment for individuals who have diuretic resistance (Kassamali and Sica, 2011). The acetamide moiety in Acetazolamide modulates electronic properties, supports hydrogen bonding, and enhances solubility, thereby optimizing the activity of the sulfonamide group responsible for carbonic anhydrase inhibition (Tsikas, 2024). Acetazolamide got its first FDA approval on 27 July 1953. It was sold under the brand name Diamox. It is chemically known as N-(5-sulfamoyl-1,3,4-thiadiazol-2-yl)acetamide. It was synthesized by reacting ammonium thiocyanate **164** with hydrazine **165** to give compound **166**, which was reacted with phosgene to give thiazole **167**. Compound **167** was reacted with acetic anhydride to give 2-acetylamino-5-mercapto-1,3,4-thiadiazole **168**, which was reacted with chlorine to give compound **169**. The target compound **170** was obtained by reacting compound **169** with ammonia (Scheme 25) (Chowdhuri, 2013).

#### 2.13.2 Role of Acetamide moiety in FDA-approved drugs

The acetamide moiety is one of the most frequently utilized functional groups in FDA-approved drugs due to its significant contribution to pharmacological activity and drug-like properties. Structurally, the acetamide group acts as both a hydrogen bond donor and acceptor, enabling strong interactions with amino acid residues within biological targets and thereby enhancing binding affinity and selectivity (Ahmad et al., 2023). In addition, the resonance-stabilised amide bond imparts conformational rigidity to drug molecules, thereby reducing entropy loss during target binding and improving receptor specificity. The incorporation of an acetamide group can increase molecular polarity and aqueous solubility without introducing a full ionic charge, thereby supporting favourable absorption, distribution, and formulation properties. For example, in Sulfacetamide, the acetamide moiety plays an important role in modulating polarity and solubility (Marchand and Nadeau, 1976). Furthermore, acetamide groups are generally metabolically stable and less basic than their corresponding amines, properties that can reduce off-target interactions and improve pharmacokinetic profiles. For example, in Trametinib, the acetamide moiety contributes to target binding while also enhancing pharmacokinetic behavior (Leonowens et al., 2014). In peptide- and protein-targeted therapeutics, the acetamide functionality can mimic a peptide bond and help stabilise bioactive conformations, thereby enhancing biological activity. For instance, in Cetrorelix and Ganirelix, the acetamide moiety contributes to maintaining structural integrity, whereas in Setmelanotide it plays an important role in facilitating receptor binding (Mannaerts 2023; Peng et al., 2026; Tur-Kaspa and Ezcurra, 2009).

## 3 Limitation and future perspective

The acetamide moiety continues to attract attention in modern medicinal chemistry due to its favorable physicochemical properties and versatility in molecular modification. In structure-based drug design (SBDD), the acetamide group’s capacity to engage in hydrogen bonding and its planar nature make it an ideal candidate for optimizing ligand target interactions, especially in kinase inhibitors and enzyme-targeting therapeutics. Acetamide substitution offers a promising strategy to enhance solubility, metabolic stability, and reduce toxicity, particularly in the development of new antibiotics and anticancer agents. These modified scaffolds can help overcome drug resistance and improve therapeutic indices. In central nervous system (CNS) drug development, the moderate lipophilicity of the acetamide group can be beneficial for blood–brain barrier permeability (Table 1). It holds future promise in designing selective monoamine oxidase (MAO) inhibitors, histone deacetylase (HDAC) inhibitors, and neuroprotective agents for disorders such as Parkinson’s and Alzheimer’s diseases. The acetamide group also holds potential in prodrug strategies, where it may be used to mask polar functional groups and enhance oral bioavailability. Enzyme-sensitive acetamide linkers can be designed to release active drug molecules selectively within pathological environments, such as tumors or sites of inflammation.

**TABLE 1:** Summary of PK/PD, Toxicity, Role of acetamide moiety and Limitations.

Sr No.	Drugs	PK/PD (key points)	Toxicity	Role of acetamide moiety	Limitation
1	Zanamivir	Inhaled NA inhibitor, low systemic absorption, acts locally in the lungs	Bronchospasm (esp. asthma)	Enhances binding to neuraminidase *via* H-bonding	Poor oral bioavailability; inhalation-only (Freund et al., 1999)
2	Oseltamivir	Oral prodrug → active carboxylate, renal excretion	Nausea, neuropsychiatric (rare)	Supports enzyme binding and stability	Resistance (NA mutations); dose adjustment in renal impairment (Batool et al., 2023)
3	Peramivir	IV NA inhibitor, long half-life	Diarrhoea, rare skin reactions	Strong binding; IV use	IV only; limited outpatient use (Witcher et al., 2019)
4	Linezolid	Oral ≈ IV bioavailability, inhibits 50S ribosome, MAO inhibition	Myelosuppression, serotonin syndrome	Improves stability, ribosomal binding	Long-term toxicity; drug interactions (Kenreigh et al., 2016)
5	Sulfacetamide	Topical, inhibits folate synthesis	Hypersensitivity, Stevens–Johnson syndrome	Modulates polarity and solubility	Resistance; allergy limits use (Scholar, 2007)
6	Acetohydroxamic acid	Oral, inhibits bacterial urease (UTIs)	Thrombosis, teratogenicity	Metal binding to urease	Narrow indication; safety concerns (Griffith et al., 1978)
7	Abarelix	Depot injection; ↓ testosterone rapidly	Allergic reactions	Structural stability	Withdrawn/limited use (Mongiat-Artus and Teillac, 2004)
8	Degarelix	SC depot, immediate testosterone suppression	Injection site reactions	Maintains conformation	Monthly injection; compliance (Doehn, 2009)
9	Trametinib	Oral, inhibits the MAPK pathway	Rash, cardiomyopathy, ocular toxicity	Contributes to binding & PK	Resistance; costly (Awada et al., 2021)
10	Etelcalcetide	IV, activates CaSR → ↓ PTH	Hypocalcemia	Controls structure and receptor binding	IV only (dialysis patients) (Yokoyama et al., 2021)
11	Apremilast	Oral, ↑ cAMP → anti-inflammatory	GI upset, depression	Stabilizes scaffold	Moderate efficacy vs. biologics (Mease et al., 2023)
12	Colchicine	Oral, inhibits microtubules	GI toxicity, myopathy	Help in tubulin binding	Narrow therapeutic index(Stamp et al., 2024)
13	Cetrorelix	SC, prevents LH surge	Injection reactions	Structural integrity	Short duration; repeated dosing (Xu et al., 2021)
14	Ganirelix	SC, similar to cetrorelix	Injection reactions	Structural integrity	Frequent dosing (Mannaerts, 2023)
15	Setmelanotide	SC, regulates appetite	Hyperpigmentation, nausea	Receptor binding	Only for rare genetic obesity(Argente et al., 2025)
16	Acetaminophen	Hepatic metabolism (glucuronidation/sulfation), central COX inhibition	Hepatotoxicity (NAPQI)	Directs metabolism; stabilizes aromatic ring	Narrow safety margin in overdose (Krenzelok and Royal, 2012)
17	Acamprosate	Renal excretion, modulates glutamate	Diarrhoea	CNS penetration, Stability	Requires TID dosing; compliance (Yahn et al., 2013)
18	N-acetylcysteine	Replenishes glutathione	Anaphylactoid reactions	Improves the stability of cysteine	Timing critical (Vilchèze and Jacobs, 2021)
19	Afamelanotide	SC implant, ↑ melanin	Hyperpigmentation	Structural role	Expensive; limited access (Minder et al., 2023)
20	Lacosamide	Oral/IV, enhances slow Na + channel inactivation	Dizziness, PR prolongation	CNS penetration; stability	Cardiac caution (Fogel et al., 2024)
21	Acetazolamide	Oral, ↓ bicarbonate reabsorption	Metabolic acidosis, hypokalemia	Enzyme binding	Weak diuretic; tolerance develops (Bradwell et al., 2014)

Despite the favorable properties of the acetamide group in medicinal chemistry, its incorporation also presents several limitations. One key concern is its potential metabolic liability; although more stable than esters, acetamide bonds can still be cleaved by amidases, leading to inactive or toxic metabolites. Additionally, the presence of the methyl group on the nitrogen can reduce hydrogen bonding capacity compared to primary amides, potentially weakening target interactions. The relatively planar and rigid structure of the amide linkage, while beneficial for receptor binding in some contexts, may also reduce conformational flexibility, limiting the ability of the molecule to adapt to dynamic binding sites. Furthermore, the acetamide moiety can increase molecular weight and polarity, which, if not balanced properly, may impair oral bioavailability or cell permeability in certain compounds (Table 1). Finally, acetamides may introduce synthetic challenges in large-scale manufacturing due to possible side reactions or low coupling efficiencies during amide bond formation.

## 4 Conclusion

The acetamide moiety is a crucial structural feature in medicinal chemistry, valued for its hydrogen bonding ability, moderate polarity, and metabolic stability. Its incorporation into drug molecules enhances pharmacokinetic and pharmacodynamic profiles, making it a versatile scaffold in various therapeutic classes. With advancements in structure-based drug design, prodrug development, CNS-targeted therapy, and synthetic methodologies, the acetamide group is poised to play an increasingly significant role in the development of safer, more effective, and environmentally sustainable pharmaceuticals. Its continued exploration will be essential for next-generation drug discovery and optimization. The acetamide moiety is present in many FDA-approved drugs. In the current work, we discuss the synthesis, use, pharmacodynamic and pharmacokinetic properties of FDA-approved drug containing an acetamide moiety, which will help to researchers to design and synthesized new generation of drugs.
